# A synonymous germline variant in a gene encoding a cell adhesion molecule is associated with cutaneous mast cell tumour development in Labrador and Golden Retrievers

**DOI:** 10.1371/journal.pgen.1007967

**Published:** 2019-03-22

**Authors:** Deborah Biasoli, Lara Compston-Garnett, Sally L. Ricketts, Zeynep Birand, Celine Courtay-Cahen, Elena Fineberg, Maja Arendt, Kim Boerkamp, Malin Melin, Michele Koltookian, Sue Murphy, Gerard Rutteman, Kerstin Lindblad-Toh, Mike Starkey

**Affiliations:** 1 Animal Health Trust, Newmarket, United Kingdom; 2 Science for Life Laboratory, Department of Medical Biochemistry and Microbiology, Uppsala University, Uppsala, Sweden; 3 Department of Clinical Sciences of Companion Animals, Utrecht University, Utrecht, The Netherlands; 4 Broad Institute of MIT and Harvard, Cambridge, MA, United States of America; 5 Veterinary Specialist Centre De Wagenrenk, Wageningen, The Netherlands; Clemson University, UNITED STATES

## Abstract

Mast cell tumours are the most common type of skin cancer in dogs, representing a significant concern in canine health. The molecular pathogenesis is largely unknown, but breed-predisposition for mast cell tumour development suggests the involvement of inherited genetic risk factors in some breeds. In this study, we aimed to identify germline risk factors associated with the development of mast cell tumours in Labrador Retrievers, a breed with an elevated risk of mast cell tumour development. Using a methodological approach that combined a genome-wide association study, targeted next generation sequencing, and TaqMan genotyping, we identified a synonymous variant in the *DSCAM* gene on canine chromosome 31 that is associated with mast cell tumours in Labrador Retrievers. *DSCAM* encodes a cell-adhesion molecule. We showed that the variant has no effect on the *DSCAM* mRNA level but is associated with a significant reduction in the level of the DSCAM protein, suggesting that the variant affects the dynamics of *DSCAM* mRNA translation. Furthermore, we showed that the variant is also associated with mast cell tumours in Golden Retrievers, a breed that is closely related to Labrador Retrievers and that also has a predilection for mast cell tumour development. The variant is common in both Labradors and Golden Retrievers and consequently is likely to be a significant genetic contributor to the increased susceptibility of both breeds to develop mast cell tumours. The results presented here not only represent an important contribution to the understanding of mast cell tumour development in dogs, as they highlight the role of cell adhesion in mast cell tumour tumourigenesis, but they also emphasise the potential importance of the effects of synonymous variants in complex diseases such as cancer.

## Author summary

The combination of various genetic and environmental risk factors makes the understanding of the molecular circuitry behind complex diseases, like cancer, a major challenge. The homogeneous nature of pedigree dog breed genomes makes these dogs ideal for the identification of both simple disease-causing genetic variants and genetic risk factors for complex diseases. Mast cell tumours are the most common type of canine skin cancer, and one of the most common cancers affecting dogs of most breeds. Several breeds, including Labrador Retrievers (which represent one of the most popular dog breeds), have an elevated risk of mast cell tumour development. Here, by using a methodological approach that combined different techniques, we identified a common inherited synonymous variant, that predisposes Labrador Retrievers to mast cell tumour development. Interestingly, we showed that this variant, despite its synonymous nature, appears to have an effect on translation dynamics as it is associated with reduced levels of DSCAM, a cell adhesion molecule. The results presented here reveal dysregulation of cell adhesion to be an important factor in mast cell tumour pathogenesis, and also highlight the important role that synonymous variants can play in complex diseases.

## Introduction

Mast cell tumours (MCTs) are the most common type of skin cancer in dogs [1], and the second most frequent form of canine malignancy in the United Kingdom [2]. Recent estimates of the mean age of dogs diagnosed with a MCT range from 7.5 to 9 years [3–5]. The majority of affected dogs are successfully treated by surgery and/or local radiotherapy, but around 30% of patients require a systemic treatment, due to tumour metastasis, and have an extremely poor prognosis [6]. Canine MCTs share many biological features with human mastocytosis [7], a heterogeneous group of neoplastic conditions characterised by the uncontrolled proliferation and activation of mast cells.

Mutations in the proto-oncogene, *c-kit*, which encodes KIT, a member of the tyrosine kinase family of receptors, are found in 20–30% of canine MCTs and in more than 90% of adult human mastocytoses [8–10]. In the case of human mastocytosis, most of the mutations are single nucleotide polymorphisms (SNPs) in exon 17, which result in alterations in the kinase domain of the receptor, with the most reported one being the V^816^D substitution [11]. In canine MCTs, most *c-kit* alterations are tandem repeats/small indels in either exons 11 and 12 (that result in alterations in the receptor’s juxtamembrane domain), or in exons 8 and 9 that encode part of the extracellular ligand-binding domain. *C-kit* alterations have recently been shown to be associated with DNA copy number alterations and with increased canine MCT malignancy [12]. They have also been explored therapeutically, and tyrosine kinase inhibitors are now used for the treatment of canine MCTs that cannot be surgically removed, or that are recurrent [13]. In the case of human mastocytosis, tyrosine kinase inhibitor resistance is associated with the most frequent *c-kit* gene mutation [14]. Although the identification of somatic *c-kit* mutations has contributed to the development of therapeutics, *c-kit* mutations are not found in the majority of canine MCTs [15].

Human mastocytosis has been associated with underlying germline risk factors [16, 17]. Pedigree dog-breeds display significant differences in the incidence of MCTs; German Shepherd Dogs, Border Collies and Cavalier King Charles Spaniels are underrepresented amongst affected dogs, while Boxers (Odds ratio: 15.11; [18]), Golden Retrievers (Odds ratio: 6.93;[18]) and Labrador Retrievers (Odds ratio: 4.63;[18]) have an increased risk of MCT development [2, 4, 18, 19]. This suggests the involvement of inherited genetic risk factors in the development of MCTs in breeds which display increased susceptibility, although there is no evidence for the occurrence of germline c-*kit* risk variants.

Certain characteristics of the domestic dog’s genome make it amenable to the genetic mapping of inherited disease-associated variants. The successive bottlenecks in the recent history of modern dog breeds, which were derived from extensive selection for phenotypic traits, have resulted in long regions of linkage disequilibrium (LD) within dog breeds [20]. The consequent reduced level of genetic complexity facilitates within-breed positional mapping of disease-associated variants, reducing the required study population size from the thousands needed for mapping human disease genes to hundreds [21].

Through a genome-wide association study (GWAS) and subsequent sequence capture and fine mapping of a region containing an associated SNP marker, Arendt and co-workers identified a germline SNP that is associated with MCTs in European Golden Retrievers [22]. The SNP is located in an exon of the Nucleotide Binding Protein (G Protein) Alpha Inhibiting Activity Polypeptide 2 (*GNAI2*) gene on canine chromosome (CFA) 20, and causes alternative exon splicing and a truncated protein [22]. In the same study, a haplotype encompassing the *HYAL4* and *SPAM11* genes on CFA14 associated with MCTs in United States (US) Golden Retrievers was also identified [22]. More recently, a GWAS identified an association between MCTs in US Labrador Retrievers and a SNP marker on CFA36 [23], although a susceptibility variant has yet to be identified,

In this work, we aimed to identify germline variants that predispose Labrador Retrievers to the development of MCTs. The identification of MCT susceptibility variants in Labrador Retrievers could not only contribute to understanding of the molecular mechanisms involved in canine MCT development, but could also help to shed light onto human mastocytosis pathogenesis. With an analysis approach that combined GWAS, targeted next generation sequencing (NGS) and TaqMan genotyping, we have identified a synonymous MCT-associated variant that is associated with significantly reduced levels of a cell adhesion molecule.

## Results

### Genome-wide association study (GWAS)

**Fig 1:**
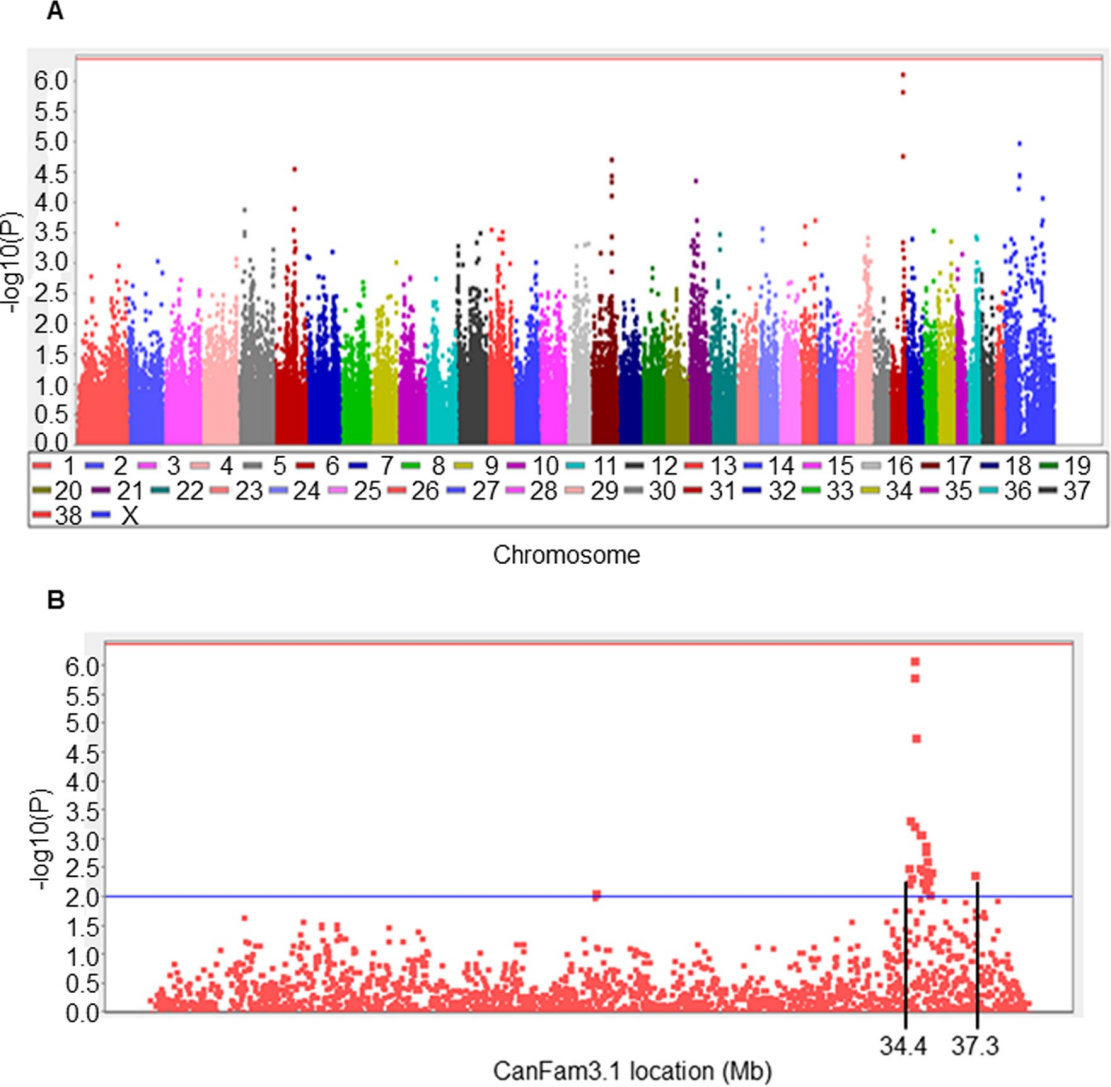
GWAS meta-analysis of MCT in Labrador Retrievers. **A.** Manhattan plot of the combined analysis of 105 cases and 85 controls from three case-control sets (Sets 1–3). Analyses comprised 115,432 SNPs. **B.** Regional association plot highlighting the regions surrounding the signal for MCT in Labrador Retrievers. The horizontal red line denotes the genome-wide association threshold based on Bonferroni correction for 115,432 tests (P-value = 4.3 x 10^−7^). The horizontal blue line represents the empirical statistical threshold used to delineate the critical region surrounding the top SNP (P-value<0.01). Plots were generated using Haploview version 4.2 [74].

We conducted an initial meta-analysis of three GWAS datasets comprising a total of 105 MCT cases and 85 controls (Sets 1, 2, and 3 in S1 Table). This analysis revealed a SNP on CFA31 that showed a strong statistical association with MCT just below the threshold of genome-wide statistical association (P-value = 7.6 x 10^−7^; Bonferroni correction for multiple testing of 115,432 SNPs: P = 4.3 x 10^−7^). The strongest associated SNP BICF2P951927 was at 34.7Mb (CanFam 3.1) (Fig 1A; S1 Fig). The common T allele at this locus was associated with an increased risk of MCT.

As MCT is likely to be a complex trait, we could not identify any clear shared haplotypes amongst cases, and examination of linkage disequilibrium (LD) amongst 2,033 GWAS SNPs on CFA31 using the pooled set of 190 dogs did not identify any other SNPs tagged by SNP BICF2P951927 at an r^2^ of 0.8 or above. We therefore delineated a critical region of association for further interrogation of the underlying sequence using a conservative empirical statistical threshold of P≤0.01 for SNP association results spanning SNP BICF2P951927 (Fig 1B). This resulted in an approximate 2.9Mb region (CanFam 3.1 co-ordinates CFA31:34433688–37366557).

Subsequent to selection of this region for resequencing, we received three additional datasets comprising in total a further 68 cases and 28 controls (Sets 4, 5 and 6 in S1 Table). We therefore repeated the above meta-analysis [one individual was dropped from dataset 3 (S1 Table) as it was reported to be suffering from cancer (not a MCT)], which comprised a total of 173 cases and 112 controls. The CFA31 association increased in strength to exceed genome-wide statistical association in this analysis (SNP BICF2P951927; P-value = 1.9 x 10^−8^; S2 Fig). We also conducted a secondary meta-analysis following individual-dataset adjustment for population stratification and the association for this SNP further increased in magnitude to P-value = 1.9 x 10^−9^; (S3 Fig). This analysis revealed additional genome-wide associated loci on other chromosomes. However, we have focused on the CFA31 region here as it showed the strongest association; analysis of the additional regions will be undertaken in future studies.

### Sequence capture and identification of candidate variants

The associated 2.9Mb region of CFA31 was captured from libraries prepared from germline DNA samples from six Labrador Retrievers affected by a MCT and six unaffected dogs over the age of 7 years, and sequenced. All the affected dogs carried two copies of the GWAS MCT-associated BICF2P951927 allele ‘*T*’, and all unaffected dogs were homozygous for the alternative allele ‘*C*’. A total of 19,930 variants (including 4,028 that were not found in any of the unaffected dogs) were identified amongst the 12 dogs. Of the variants, 126 displayed the same segregation pattern as the GWAS MCT-associated SNP (i.e. the six cases were homozygous for the reference allele, and the six controls were homozygous for an alternative allele). However, all 126 variants were located within introns (that were part of a single gene, *DSCAM*), and these were not considered to be strong candidate MCT susceptibility variants. Alternatively, variants were selected for further analysis on the basis of a combination of both: (a) The potential functional consequence assessed according to the position of a variant (regardless of whether the variant was predicted, by Variant Effect Predictor and/or SIFT, to be deleterious), and (b) The extent to which a variant segregated between the six cases and six controls. Specifically, 23 variants (22 SNPs and one deletion; Tables 1 and 2) that fulfilled both of the following criteria were selected for genotyping in a large case-control set:

1. Locus position: exon, including UTRs, and predicted to be deleterious or non-deleterious, OR splice region
                                                                  AND
2. Segregation: One allele is present as at least one copy in at least one case and is not present in any of the controls [i.e. (a) Biallelelic loci: one allele can be present in both cases and controls, but the second allele must be unique to the cases; (b) Multi-allele loci: multiple alleles can be present in both cases and controls, but one allele must be unique to the cases)

**Table 1:** CFA31 germline variants selected from resequencing data for further investigation.

dbSNP ID.	CFA31 base co-ordinate (CanFam3.1)	Gene	Gene DNA Strand	Variant location	Variant type	Variant consequence (VEP)	SIFT Predicted Impact
rs852630575	34482267	*IGSF5*	Plus	3 prime UTR	SNP	3 prime UTR	N/A
Not available	34482866	*IGSF5*	Plus	3 prime UTR	SNP	3 prime UTR	N/A
N/A	34667505	*DSCAM*	Minus	3 prime UTR	indel	3 prime UTR	N/A
rs850678541	34760750	*DSCAM*	Minus	exon	SNP	Synonymous	N/A
rs852645717	34760777	*DSCAM*	Minus	exon	SNP	Synonymous	N/A
rs23691670	34962258	*DSCAM*	Minus	exon	SNP	Synonymous	N/A
Not available	35933663	*TMPRSS2*	Minus	3 prime UTR	SNP	3 prime UTR	N/A
rs852502899	36248583	*PRDM15*	Minus	exon	SNP	Synonymous	N/A
rs852865506	36295441	*C2CD2*	Minus	exon	SNP	Missense	tolerated
rs850678905	36309240	*C2CD2*	Minus	splice region and intron	SNP	splice region and intron	N/A
rs850787912	36488364	*UMODL1*	Plus	exon	SNP	Synonymous	N/A
rs852234331	36670892	*TFF2*	Minus	5 prime UTR	SNP	5 prime UTR	N/A
rs851262732	36710190	*TMPRSS3*	Minus	exon	SNP	missense	deleterious
rs851939503	36710860	*TMPRSS3*	Minus	exon/intron (transcript difference)	SNP	intron + missense	N/A + low confidence deleterious
rs851463252	36715967	*TMPRSS3*	Minus	exon and splice region	SNP	Synonymous	N/A
rs852977026	36716021	*TMPRSS3*	Minus	exon	SNP	Synonymous	N/A
rs852791601	36718391	*TMPRSS3*	Minus	splice region and intron	SNP	N/A	N/A
rs850730691	36729172	*TMPRSS3*	Minus	intron [TMPRESS-202] and exon [TMPRSS-201]	SNP	splice region and intron	N/A
rs852281859	36741129	*UBASH3A*	Plus	exon	SNP	Synonymous	N/A
rs852901066	36889622	*SLC37A1*	Plus	3 prime UTR	SNP	3 prime UTR	N/A
rs852902530	37034944	*PDE9A*	Plus	exon	SNP	Missense	tolerated
rs852645838	37214961	*PKNOX1; ENSCAFG00000010539; NDUFV3*	Plus; Minus; Plus	splice region and intron; intron; intron	SNP	splice region and intron; intron; intron	N/A
Not available	37278660	*U2AF1; ENSCAFG00000029964*	Minus; Plus	Upstream; exon	SNP	upstream + missense	N/A + low confidence deleterious

The dbSNP database [24] ID of a previously reported SNP is stated where available. The consequence of each variant was predicted using SIFT [25]. N/A = not applicable.

**Table 2:** Genotypes of 12 resequenced Labrador Retrievers at selected CFA31 candidate MCT susceptibility loci.

dbSNP ID.	CFA31 base co-ordinate (CanFam3.1)	Reference allele (a)	Alternative (‘variant’) alleles (b, c)	Genotypes	
				No. MCT cases	No. Controls
rs852630575	34482267	*G*	*A*	2 x *a/a*; 4 x *a/b*; 0 x *b/b*	6 x *a/a*; 0 x *a/b*; 0 x *b/b*
Not available	34482866	*C*	*G*	2 x *a/a*; 4 x *a/b*; 0 x *b/b*	6 x *a/a*; 0 x *a/b*; 0 x *b/b*
N/A	34667505	*AACACACAC*	*AAC*, *AACACAC*	0 x *a/a*; 0 x *a/b*; 0 x *a/c*; 6 x *b/b;* 0 x *b/c;* 0 x *c/c*	0 x *a/a*; 0 x *a/b;* 1 x *a/c*; 0 x *b/b;* 0 x *b/c*; 5 x *c/c*
rs850678541	34760750	*G*	*A*	1 x *a/a*; 3 x *a/b*; 2 x *b/b*	6 x *a/a*; 0 x *a/b*; 0 x *b/b*
rs852645717	34760777	*G*	*A*	2 x *a/a*; 4 x *a/b*; 0 x *b/b*	6 x *a/a*; 0 x *a/b*; 0 x *b/b*
rs23691670	34962258	*G*	*A*	2 x *a/a*; 2 x *a/b*; 2 x *b/b*	6 x *a/a*; 0 x *a/b*; 0 x *b/b*
Not available	35933663	*C*	*T*	3 x *a/a*; 3 x *a/b*; 0 x *b/b*	6 x *a/a*; 0 x *a/b*; 0 x *b/b*
rs852502899	36248583	*G*	*A*	2 x *a/a*; 3 x *a/b*; 1 x *b/b*	0 x *a/a*; 0 x *a/b*; 6 x *b/b*
rs852865506	36295441	*C*	*T*	2 x *a/a*; 3 x *a/b*; 1 x *b/b*	6 x *a/a*; 0 x *a/b*; 0 x *b/b*
rs850678905	36309240	*A*	*G*	1 x *a/a*; 4 x *a/b*; 1 x *b/b*	6 x *a/a*; 0 x *a/b*; 0 x *b/b*
rs850787912	36488364	*C*	*T*	1 x *a/a*; 4 x *a/b*; 1 x *b/b*	6 x *a/a*; 0 x *a/b*; 0 x *b/b*
rs852234331	36670892	*G*	*A*	2 x *a/a*; 3 x *a/b*; 1 x *b/b*	6 x *a/a*; 0 x *a/b*; 0 x *b/b*
rs851262732	36710190	*C*	*T*	2 x *a/a*; 3 x *a/b*; 1 x *b/b*	6 x *a/a*; 0 x *a/b*; 0 x *b/b*
rs851939503	36710860	*G*	*A*	2 x *a/a*; 3 x *a/b*; 1 x *b/b*	6 x *a/a*; 0 x *a/b*; 0 x *b/b*
rs851463252	36715967	*A*	*G*	1 x *a/a*; 4 x *a/b*; 1 x *b/b*	6 x *a/a*; 0 x *a/b*; 0 x *b/b*
rs852977026	36716021	*G*	*A*	2 x *a/a*; 3 x *a/b*; 1 x *b/b*	6 x *a/a*; 0 x *a/b*; 0 x *b/b*
rs852791601	36718391	*A*	*G*	1 x *a/a*; 4 x *a/b*; 1 x *b/b*	6 x *a/a*; 0 x *a/b*; 0 x *b/b*
rs850730691	36729172	*A*	*G*	2 x *a/a*; 3 x *a/b*; 1 x *b/b*	6 x *a/a*; 0 x *a/b*; 0 x *b/b*
rs852281859	36741129	*C*	*T*	2 x *a/a*; 3 x *a/b*; 1 x *b/b*	6 x *a/a*; 0 x *a/b*; 0 x *b/b*
rs852901066	36889622	*A*	*G*	2 x *a/a*; 3 x *a/b*; 1 x *b/b*	6 X *a/a*; 0 x *a/b*; 0 x *b/b*
rs852902530	37034944	*A*	*G*	2 x *a/a*; 3 x *a/b*; 1 x *b/b*	6 x *a/a*; 0 x *a/b*; 0 x *b/b*
rs852645838	37214961	*G*	*A*	1 x *a/a*; 3 x *a/b*; 2 x *b/b*	6 x *a/a*; 0 x *a/b*; 0 x *b/b*
Not available	37278660	*C*	*T*	2 x *a/a*; 3 x *a/b*; 1 x *b/b*	6 x *a/a*; 0 x *a/b*; 0 x *b/b*

The reference and alternative alleles shown refer to nucleotide bases in the plus DNA strand. N/A = not applicable.

### Candidate MCT susceptibility variants—Association analysis in a larger case-control set

TaqMan Genotyping Assays were designed for the 22 SNPs. The indel variant at CFA31:34667505 was genotyped by fluorescent end point PCR fragment analysis. The 23 candidate MCT susceptibility loci were genotyped in 407 UK Labrador Retrievers comprising 191 MCT cases and 216 controls (including 71 cases and 42 controls from the GWAS study) (S2 Table). The SNP rs850787912 was excluded from the association analysis because it strongly deviated from Hardy-Weinberg distribution (P-value = 2.2 x 10^−83^), indicating assay failure.

One of the 22 analysed loci (SNP rs850678541, at CFA31:34760750) demonstrated statistical association with MCT (P-value = 5.2 x 10^−4^; Table 3). This association was stronger than that of the strongest associated GWAS SNP BICF2P951927 (Table 4). The SNP is associated with MCT with an odds ratio of 1.67 (95% confidence interval 1.24–2.24), and explains 2% (pseudo r^2^) of the MCT trait in this breed. The alternative ‘*A’* allele is common—72% of the genotyped dogs (including 67% of controls) carried at least one copy, and 25% of the dogs (including 20% of controls) carried two copies (Table 4). This allele increases the risk of MCT development by 1.66 x (ratio of heterozygote odds: reference allele homozygote odds; 95% confidence interval 0.99–2.77) when present as one copy, and by 2.79 x (ratio of alternative allele homozygote odds: reference allele homozygote odds; 95% confidence interval 1.55–5.03) when present as two copies.

**Table 3:** Association analysis results for selected CFA31 candidate MCT susceptibility variants.

dbSNP ID.	CFA31 base co-ordinate (CanFam3.1)	No. MCT cases	No. Controls	P-value
rs852630575	34482267	189	213	0.25
Not available	34482866	184	213	0.61
N/A	34667505	172	198	0.16/0.27*
rs850678541	34760750	168	194	5.2 x 10^−4^
rs852645717	34760777	181	214	0.06
rs23691670	34962258	186	207	0.52
Not available	35933663	177	202	0.28
rs852502899	36248583	173	194	0.76
rs852865506	36295441	184	206	0.43
rs850678905	36309240	185	206	0.98
rs852234331	36670892	180	202	0.44
rs851262732	36710190	180	203	0.34
rs851939503	36710860	163	167	0.91
rs851463252	36715967	181	204	0.72
rs852977026	36716021	154	142	0.66
rs852791601	36718391	172	201	0.89
rs850730691	36729172	178	204	0.64
rs852281859	36741129	182	204	0.60
rs852901066	36889622	181	199	0.57
rs852902530	37034944	179	195	0.87
rs852645838	37214961	174	196	0.80
Not available	37278660	120	168	0.49

Detailed is the number of cases and controls genotyped for each indicated variant, and the P-value obtained by testing for association using logistic regression and log likelihood ratio test. Bonferroni correction for testing: P = 2.3 x 10^−3^.

*The test for association between the indel at CFA31: 34667505 and MCT was done for both genotypes/alleles using Fisher’s exact test, due to its multiallelic nature. The numbers of MCT and control dogs genotyped in each assay varied due to DNA sample availability and variable assay performance.

**Table 4:** Genotypes of the strongest associated GWAS SNP and SNP rs850678541 in Labrador Retrievers.

**Top GWAS SNP BICF2P951927 and SNP rs850678541 genotypes of LR GWAS set**					
**BICF2P951927**	**No. Cases**	**No. Controls**	**Odds ratio** **(95% CI)**	**P-value**	**Model fit** **(pseudo r**^**2**^**)**
*C*/*C*	1	3	1.98 (0.90–4.36)	0.08	
*C*/*T*	21	14			0.03
*T*/*T*	29	12			
**rs850678541**			3.00 (1.45–6.18)	1.4 x 10^−3^	
*G*/*G*	8	12			0.10
*G*/*A*	24	14			
*A*/*A*	19	3			
**SNP rs850678541 genotypes of an extended LR case-control set**					
*G*/*G*	34	65	1.67 (1.24–2.24)	5.2 x 10^−4^	
*G*/*A*	80	92			0.02
*A*/*A*	54	37			

CI: confidence interval. The P-values presented were obtained by logistic regression and log likelihood ratio tests. The Labrador Retriever (LR) GWAS subset given in this table contains only dogs for which both BICF2P951927 and rs850678541 genotypes were available. N/A = not applicable.

### Investigation of the biological effects of the alternative allele of SNP rs850678541

**Fig 2:**
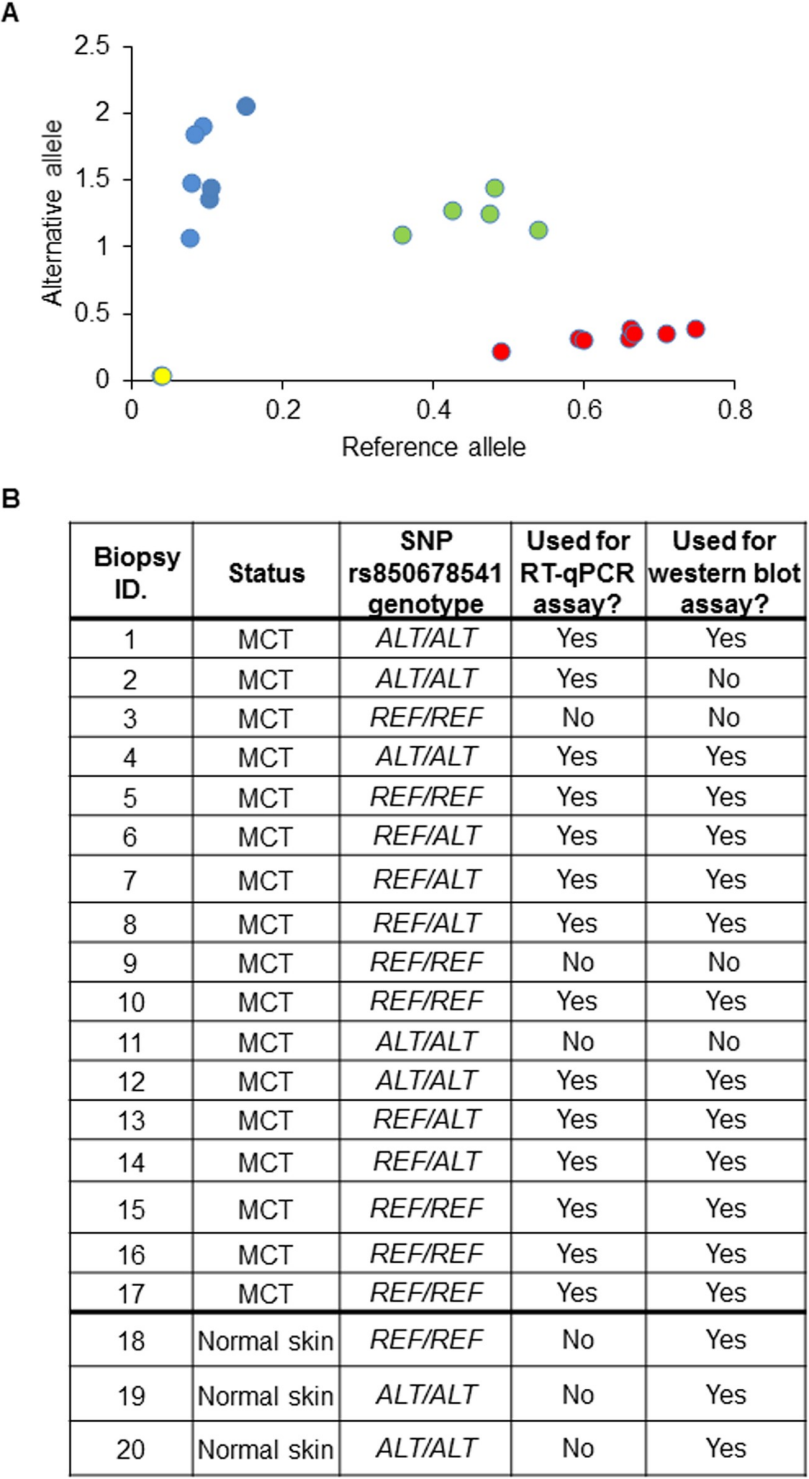
SNP rs850678541 genotyping of Labrador Retriever tissue biopsies. **A.** Allelic discrimination plot, generated by the TaqMan Genotyper Software, showing the distribution of the 20 biopsies analysed. Reference allele: *G*, and alternative allele: *A*. The SNP rs850678541 genotypes are represented by: blue spheres—Alternative (variant) ‘*A*’ allele homozygote; red spheres—Reference ‘*G*’ allele homozygote; green spheres—*G/A* heterozygote. **B.** Table showing the status and SNP rs850678541 genotype of each biopsy.

The alternative (variant) allele of SNP rs850678541 (CFA31:34760750) represents a G>A transition (plus DNA strand) located in exon 16 of the canine *DSCAM* gene, which encodes a cell adhesion molecule. It occurs in the third base of a codon (representing arginine), and, as such, is a synonymous mutation (changing the codon from *CGC* to *CGT*). A growing body of evidence indicates that, although synonymous mutations do not cause amino acid sequence changes, they can have an effect on factors such as mRNA stability and translation kinetics, and thus have significant biological consequences [26–30]. Consequently, we investigated if the alternative allele of SNP rs850678541 had any effect on *DSCAM* mRNA and protein levels. DNA, RNA and protein were simultaneously extracted from 17 RNAlater-preserved MCT biopsies borne by Labrador Retrievers (representative of the three locus CFA31:34760750 genotypes; Biopsies #1–17 in Fig 2), and from normal skin biopsies from three Labrador Retrievers (Biopsies #18–20 in Fig 2). The levels of *DSCAM* mRNA and protein expression were compared between the three genotypes.

#### RT-qPCR assay of DSCAM mRNA expression

Three sub-optimally ‘low concentration’ MCT RNA samples were excluded availing 14 of the 17 MCT biopsy RNA samples for assay of *DSCAM* expression. Each MCT RNA sample belonged to one of three genotype groups: (a) homozygous for SNP rs850678541 reference ‘*G*’ allele, (b) homozygous for SNP rs850678541 alternative ‘*A*’ allele, and (c) heterozygous. Prior to RT-qPCR analysis, cDNAs prepared from the 14 available MCT RNAs were screened for the presence of PCR inhibitors using the SPUD assay, since the PCR inhibitor heparin is commonly found in mast cells [31]. The mean SPUD amplicon Cq value and Cq SD measured for each MCT cDNA are presented in S3 Table. As the SPUD amplicon mean Cq value showed little variation across the 14 MCT cDNAs assayed (Cq SD = 0.24) and the largest difference between the mean SPUD Cq value for any two of the three genotype groups was 0.21, differences in the levels of PCR inhibitors present in each MCT sample were considered to be negligible and all 14 MCT cDNAs were used for *DSCAM* mRNA analysis.

**Fig 3:**
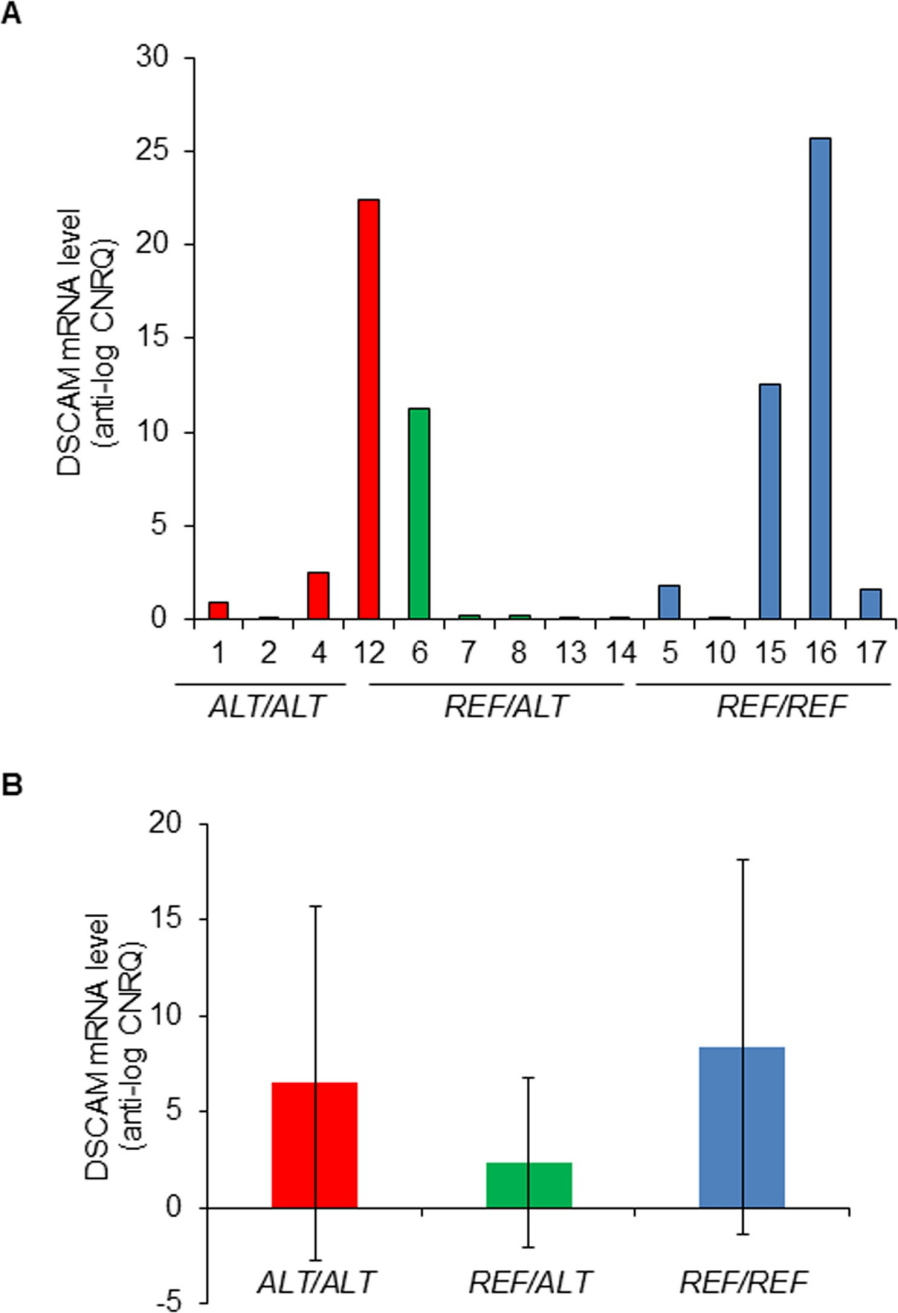
*DSCAM* mRNA levels in MCTs from Labrador Retrievers with different SNP rs850678541 genotypes. **A.** Bar charts showing the *DSCAM* mRNA level (anti-log of the CNRQ—Calibrated Normalised Relative Quantity—values) of the indicated biopsies, grouped by their SNP rs850678541 genotype. The differences between the groups (as assessed by Kruskal-Wallis and Mann-Whitney U tests, respectively) were not statistically significant. **B.** Bar charts showing the mean *DSCAM* mRNA level (anti-log of the CNRQ values) for each genotype group. Error bars represent standard deviations. The SNP rs850678541 genotypes are represented by: *ALT/ALT*—Alternative (variant) ‘A’ allele homozygote; *REF/REF*—Reference ‘G’ allele homozygote; *REF/ALT*—GA heterozygote.

RT-qPCR assay of *DSCAM* mRNA expression targeted a 124 bp fragment in exon 16 of the *DSCAM* gene (ENSCAFG00000010139, which encodes a 7,725b transcript ENSCAFT00000016117). The difference between the *DSCAM* mRNA levels (S4 Table) measured for the three genotype groups (Fig 3) was not statistically significant (P = 0.32; Kruskal-Wallis test) (Fig 3A). Similarly, pairwise comparisons between genotype groups indicated no statistically significant difference in the *DSCAM* mRNA levels (Reference allele homozygotes v heterozygotes: Mann-Whitney U test P-value = 0.15; alternative allele homozygotes v heterozygotes: Mann-Whitney U test P-value = 0.14; reference allele homozygotes v alternative allele homozygotes: Mann-Whitney U test P-value = 1.0).

#### Western blot assay of DSCAM protein expression

**Fig 4:**
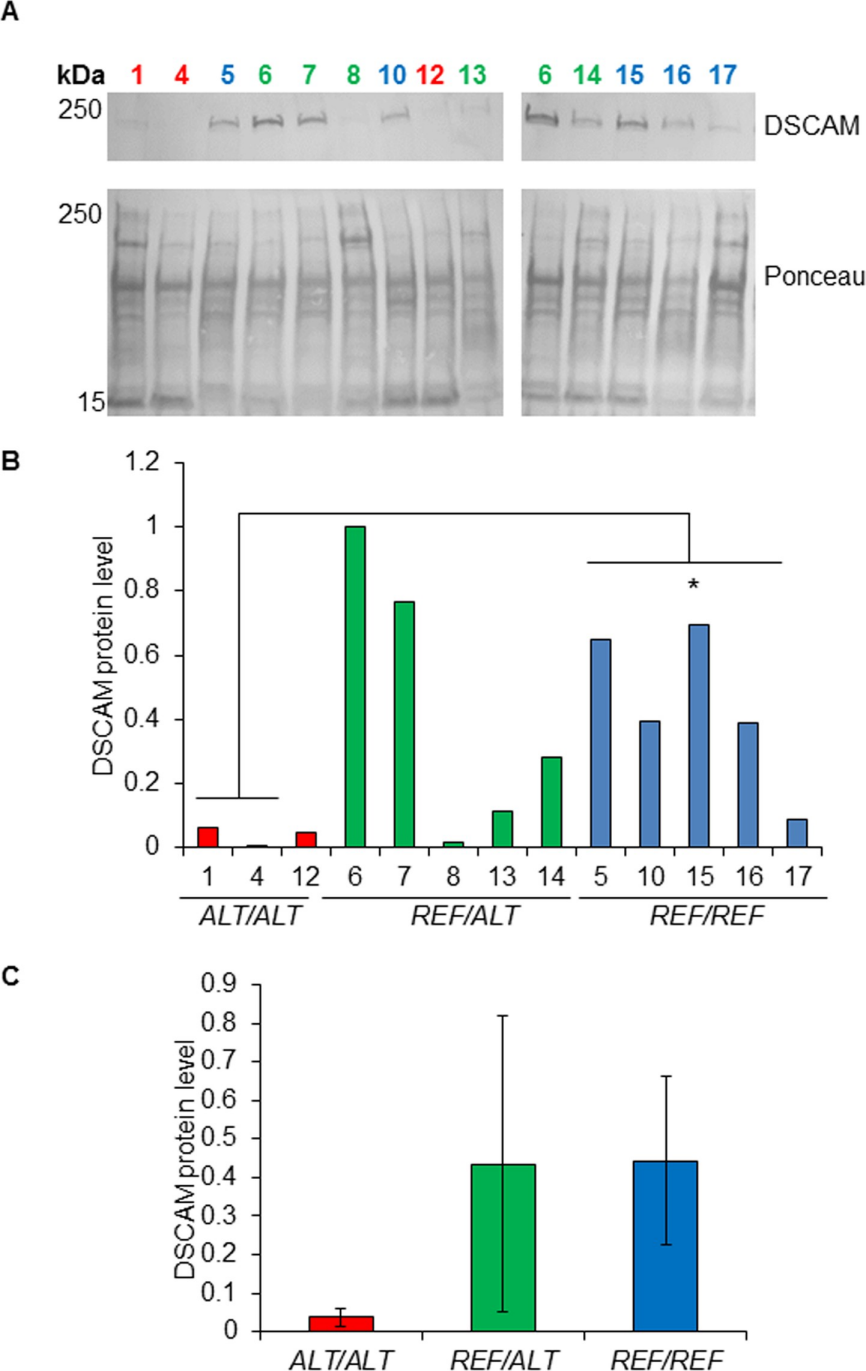
DSCAM protein levels in MCTs from Labrador Retrievers with different SNP rs850678541 genotypes. **A.** DSCAM western blot prepared using protein samples extracted from the indicated MCT biopsies. Ponceau S total protein staining was used for normalisation to adjust for variation in protein loading. Sample number colours indicate the SNP rs850678541 genotype: Red = *Alt/Alt* [Alternative (variant) ‘A’ allele homozygote]; Green = *Ref/Alt* [GA heterozygote]; Blue = *Ref/Ref* [Reference ‘G’ allele homozygote]. **B.** Bar charts showing the DSCAM protein levels of the indicated biopsies, grouped by their SNP rs850678541 genotype, as quantified by the Image Lab software (using Ponceau S-measured total protein quantity as the normaliser, and ‘Sample 6’ as the inter-membrane calibrator). *Mann-Whitney two-tailed U test P = 0.04. **C.** Bar charts showing the mean DSCAM protein level of each SNP rs850678541 genotype group. Error bars represent standard deviations.

**Fig 5:**
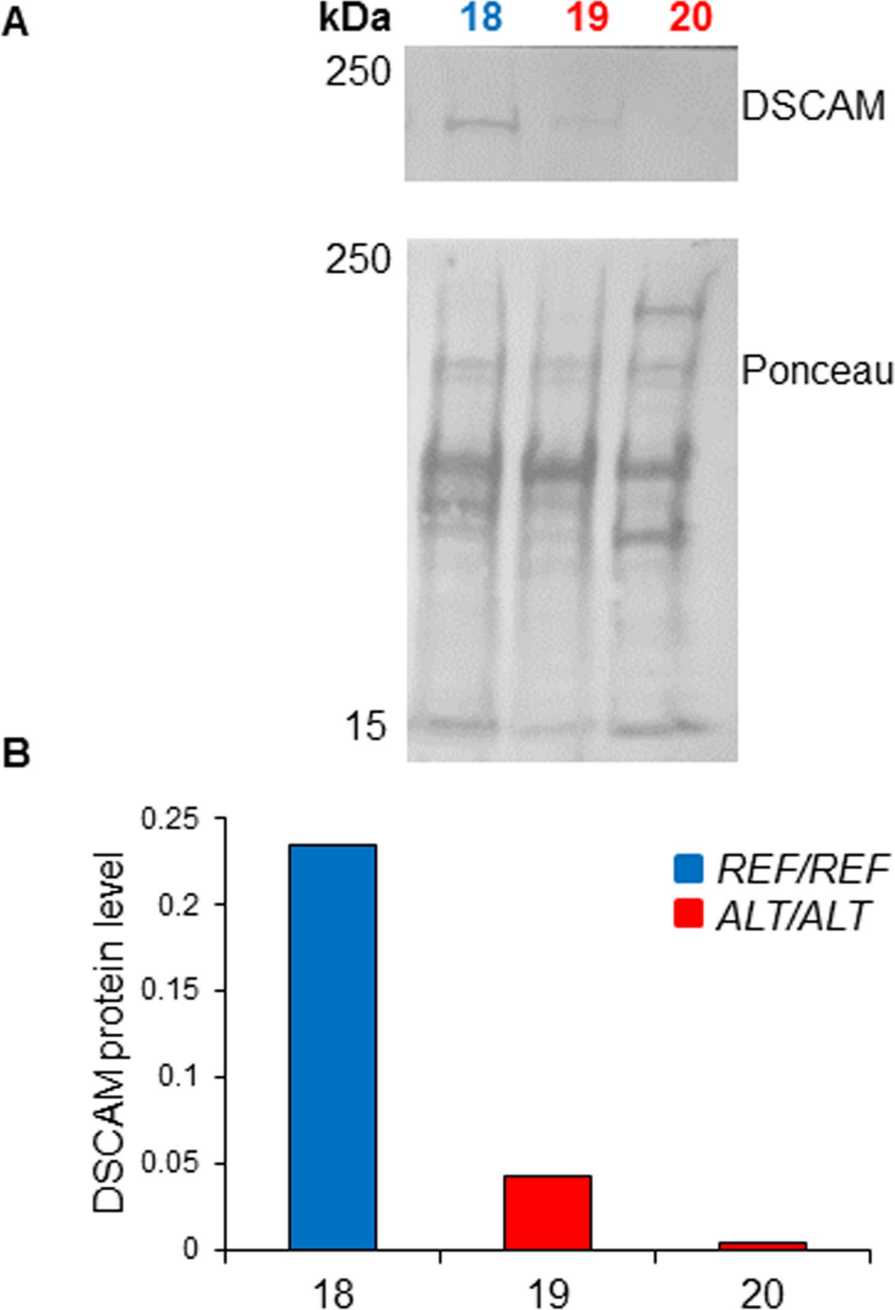
**DSCAM protein levels in normal skin biopsies from Labrador Retrievers with different SNP rs850678541 genotypes: A.** DSCAM protein levels in normal skin biopsies from Labrador Retrievers with different SNP rs850678541 genotypes: A. DSCAM Western blot prepared using protein samples extracted from the indicated skin biopsies (Fig 2B IDs: 18, 19, 20). Ponceau S total protein staining was used for normalisation to adjust for variation in protein loading. Sample number colours indicate the SNP rs850678541 genotype: Red = *Alt/Alt* [Alternative (variant) ‘A’ allele homozygote]; Blue = *Ref/Ref* [Reference ‘G’ allele homozygote]. **B.** Histogram showing the DSCAM protein levels of the indicated biopsies, as quantified by the Image Lab software (using Ponceau S-measured total protein quantity as the normaliser, and ‘Sample 6’ as the inter-membrane calibrator).

The level of DSCAM protein in 13 MCT biopsies was measured by semi-quantitative western blot (S5 Table). Four of the 17 MCT biopsies were excluded from this analysis on the basis of their total protein staining pattern, which indicated degradation (S4 Fig). Each MCT protein sample belonged to one of three genotype groups: (a) homozygous for the SNP rs850678541 reference ‘*G*’ allele, (b) homozygous for the SNP rs850678541 alternative ‘*A*’ allele, and (c) heterozygous. A substantial degree of variability in the DSCAM protein level was observed between biopsies borne by dogs that were heterozygous for SNP rs850678541 (Fig 4), but the difference between the DSCAM protein levels measured for the three genotype groups was not statistically significant (P = 0.09; Kruskal-Wallis test) (Fig 4B). Differences between the DSCAM protein levels of the homozygous reference allele group and the heterozygote group (Mann-Whitney U test P-value = 1.0), and between the homozygous alternative allele group and heterozygotes (Mann-Whitney U test P-value = 0.14) were not statistically significant. However, the difference between the DSCAM protein expression levels of the reference allele homozygotes and alternative allele homozygotes was statistically significant (Mann-Whitney U test P-value = 0.04; Fig 4B). The mean level of DSCAM protein in the alternative allele homozygous MCT biopsies was approximately ten times lower than that in the reference allele homozygotes (Fig 4C). The same result was obtained regardless of whether normalisation for variable protein loading was performed using total detected protein measured by Ponceau (Fig 4), or by Stain-Free technology (S5 Fig). A similar large-fold difference between the levels of DSCAM protein expression detected for reference allele and alternative allele homozygotes was observed for three normal skin biopsies analysed (Fig 5).

### Evaluation of the possibility that a variant at a locus in LD with SNP rs850678541 could cause alternative splicing resulting in the ten-fold reduction in DSCAM protein expression observed in SNP rs850678541 alternative allele homozygotes

**Fig 6:**
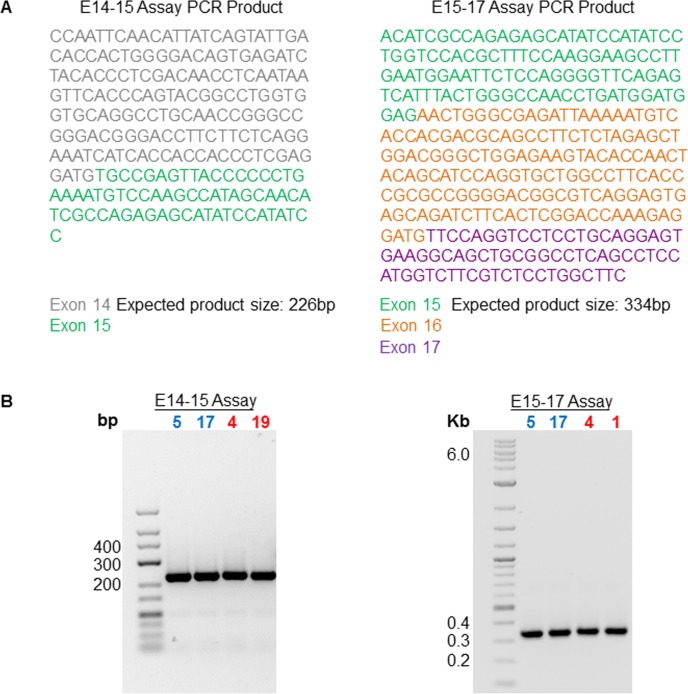
**Assay for *DSCAM* alternative splicing: A.** PCR amplicons derived from 7,725b *DSCAM* transcript *ENSCAFT00000016117* expected to be amplified by the E14-15 and E-15-17 Assays. The *DSCAM* exonic sequences illustrated are minus DNA strand sequences. **B.** Gel Image showing the PCR products obtained by running the indicated PCR assays on MCT cDNAs. The sample numbers represent the IDs. of the MCT biopsies from which RNA was isolated. Sample number colours indicate the SNP rs850678541 genotype: Red = *Alt/Alt* [Alternative (variant) ‘*A*’ allele homozygote]; Blue = *Ref/Ref* [Reference ‘*G*’ allele homozygote].

As SNP rs850678541 is a synonymous variant, we investigated the possibility that it was not a causal variant, but that it tagged another *DSCAM* gene variant that actually caused the observed protein level effect. The variants identified by targeted resequencing of the associated 2.9Mb CFA31 region in 12 Labrador Retrievers included 2,045 at loci in the *DSCAM* gene. In addition to SNP rs850678541, of the remaining 2,044 *DSCAM* gene variants, 13 were located in exons (five synonymous variants and eight in the 3'-UTR), 1975 were located in introns (including one within a ‘splice region’), and 56 were upstream of the *DSCAM* gene. Consequently, we screened for LD between SNP rs850678541 and each of the remaining 2,044 loci (1,950 biallelic and 94 multiallelic). Twenty-two intronic *DSCAM* loci (comprising 13 SNPs and nine indels) were found to be in LD with SNP rs850678541 at an r^2^ of 0.8 (S6 Table). Intronic variants can disrupt splicing enhancer sites or branch points, and can also activate cryptic splicing sites [32] that compete with the canonical sites, leading to the generation of alternative splicing products [33]. The antibody employed in Western blot analysis recognises an epitope that is translated from a sequence located in exon 23 of the *DSCAM* gene. Consequently, an intronic mutation that generates an alternative mRNA transcript lacking exon 23 would not necessarily be detectable by RT-qPCR assay of *DSCAM* exon 16 expression, but could lead to a reduction in the level of the 196kDa protein encoded by the 30 exon 1,7725b *DSCAM* mRNA transcript (*ENSCAFT00000016117*), such as that observed in the MCT and normal skin biopsies homozygous for the SNP rs850678541 alternative ‘*A*’ allele (Figs 4 and 5). The 22 intronic variants were screened for those that could potentially affect mRNA splicing using the Human Splicing Finder web tool [34]. This analysis identified three variants that could potentially lead to the generation of new splicing products: (1) a variant (at CFA31:34767321; biallelic locus ‘16’ in S6 Table) in the intron between exons 14 and 15 that could disrupt the splicing branch point, and generate a splicing product that would include 73 additional nucleotides from the intron; (2) a variant (at CFA31:34761118; biallelic locus ‘8’ in S6 Table) in the intron between exons 15 and 16 that could activate a cryptic intronic donor splice site that (if used instead of the canonical site) would generate a splicing product including 5,980 nucleotides from the intron; and (3) a variant (at CFA31:34760052; biallelic locus ‘18’ in S6 Table) in the intron between exons 16 and 17 that could also activate a cryptic intronic donor splice site that (if used) would generate a splicing product with an additional 644 nucleotides from the intron. End point PCR assays were performed to investigate if any of the three predicted alternative splice variants were present in MCT biopsies borne by dogs homozygous for the alternative allele ‘*A*’ of SNP rs850678541, on the presumption that dogs homozygous for this allele would also be homozygous for the variants at the three intronic loci shown to be in LD with SNP rs850678541. The possible effect of the variant located in the intron between exons 14 and 15 was investigated using an assay (E14-15 Assay) that targets an amplicon spanning the end of exon 14 and the beginning of exon 15, whilst an assay (E15-17 Assay) targeting an amplicon spanning the end of exon 15, exon 16, and the beginning of exon 17 was employed to assess the possible effects of the variants located in the introns between exons 15 and 16, and between exons 16 and 17, respectively. End point PCR assay of MCT cDNAs prepared from two SNP rs850678541 reference ‘*G*’ allele homozygotes and two SNP rs850678541 alternative allele ‘*A*’ homozygotes showed no differences between the exonic fragments amplified (Fig 6). For both the E14-15 and E15-17 Assays only the expected exonic mRNA fragment was amplified irrespective of SNP rs850678541 genotype (Fig 6). These results indicate that the variants at the three intronic *DSCAM* loci in LD with SNP rs850678541 are not likely to cause the ten-fold reduction in DSCAM protein expression observed in MCTs and normal skin tissues that are homozygous for SNP rs850678541 alternative allele ‘*A*’.

### Is the SNP rs850678541 genotype associated with the age of MCT development and MCT metastasis?

We investigated if the SNP rs850678541 genotype was associated with a difference in the mean age at which a Labrador Retriever developed a MCT. Labrador Retrievers which were homozygous for the reference ‘*G*’ allele had a later mean age of onset (8.59 ± 2.75 years; n = 54) than heterozygotes (7.81 ± 2.74 years; n = 69) and dogs homozygous for the alternative ‘*A*’ allele (7.82 ± 2.92 years; n = 25). However, the differences between the three genotypes (Kruskal-Wallis test P-value = 0.52), and between pairs of genotypes (e.g. reference allele homozygotes v alternative allele homozygotes: Mann-Whitney U test P-value = 0.37) were not statistically significant. As the SNP rs850678541 alternative allele is associated with a significant reduction in the protein level expression of a cell adhesion molecule, we also undertook a preliminary investigation of whether it is also associated with MCT metastasis in Labrador Retrievers. The SNP was genotyped in five Labrador Retrievers that died due to MCT metastatic disease (as confirmed by abdominal/thoracic imaging and lymph node histopathological examination) and eight Labrador Retrievers for which MCT metastases could not be detected and whom were still alive 1,000 days post-diagnosis. The dogs genotyped were either heterozygotes (ten dogs: five with metastatic MCT, and five with non-metastatic MCT), or homozygous for the reference ‘*G*’ allele (three dogs with non-metastatic MCT). No association was found between MCT metastasis and the SNP rs850678541 genotype (Fisher exact test P-value = 0.43) in this small preliminary dataset.

### The SNP rs850678541 alternative allele is also associated with MCT development in Golden Retrievers

SNP rs850678541 was genotyped in a MCT case-control set of UK Golden Retrievers, a breed that is both closely related to Labrador Retrievers [35] and has an elevated risk of developing MCTs [2, 4, 19]. Germline DNAs from 37 Golden Retrievers that either currently or previously had a MCT and 53 dogs aged at least 7 years of age that had never been affected by any form of cancer were genotyped. SNP rs850678541 demonstrated statistical association with MCT (P-value = 0.01) that was directionally consistent and of a similar magnitude of effect to that observed in Labrador Retrievers, and accounted for 5% (pseudo r^2^) of the MCT trait in Golden Retrievers (Table 5). The alternative ‘*A*’ allele was common in this Golden Retriever set (70% of the dogs, including 62% of controls, carried at least one copy, and 26% of the dogs, including 17% of controls, carried two copies) (Table 5). This allele increases the risk of MCT development by 1.90 x (ratio of heterozygote odds: reference allele homozygote odds; 95% confidence interval 0.65–5.54) when present as one copy, and by 4.44 x (ratio of alternative allele homozygote odds: reference allele homozygote odds; 95% confidence interval 1.34–14.77) when present as two copies.

**Table 5:** The association between SNP rs850678541 and/or SNP rs851590509 and MCT in a set of Golden Retrievers.

genotypes	No. MCT cases	No. controls	Odds ratio (95% CI)	P-value	Model fit (pseudo r^2^)
**SNP rs850678541**					
G/G	7	20	2.11 (1.16–3.86)	0.01	
G/A	16	24			0.05
A/A	14	9			
**SNP rs851590509**					
G/G	1	14	6.82 (2.91–16.00)	1.5 x 10^−7^	
G/A	9	28			0.23
A/A	27	11			
**Combined Analysis**					
N/A	37	53	8.04 (3.17–20.43)	2.6 x 10^−8^	
					0.29

			

CI: confidence interval. N/A: not applicable. The P-values presented were obtained by logistic regression and log likelihood ratio test.

SNP rs850678541 was also genotyped in the Border Collie (110 dogs) and Cavalier King Charles Spaniel (105 dogs), two breeds which are under-represented amongst dogs that develop MCTs [4, 19]. The alternative ‘*A*’ allele was present in both breeds at a frequency (Border Collie: 0.058; Cavalier King Charles Spaniel: 0.38) lower than that in the Labrador Retriever (0.49) and Golden Retriever (0.48).

### The Golden Retriever MCT susceptibility SNP rs851590509 in *GNAI2* is rare in Labrador Retrievers

We investigated if the MCT susceptibility SNP rs851590509 at CFA20: 39080161, which was previously identified in European Golden Retrievers by Arendt and co-workers [22], is also associated with MCTs in Labrador Retrievers. The variant is located in an exon of the *GNAI2* gene and causes alternative exon splicing and a truncated protein. We performed TaqMan genotyping of rs851590509 in 167 cases and 193 controls from our extended MCT case-control set of UK Labradors. The alternative ‘*A*’ ‘risk allele’ of the SNP is rare in Labrador Retrievers (frequency in the whole set: 0.007), and no association was found with MCTs (Fisher Exact P-value = 0.09). Arendt *et al*. also identified a putative MCT susceptibility locus at CFA14:14.7Mb in Golden Retrievers from the United States (although the most significantly associated SNPs were not found to be associated with the MCT trait in European Golden Retrievers). A causal variant for the CFA14:14.7Mb association has yet to be identified, and for this reason we did not screen for associations between CFA14:14.7Mb SNPs and the MCT trait in our UK Labrador Retriever cohort.

### Combined analysis of the rs850678541 and rs851590509 variants and risk of MCT in Golden Retrievers

Our next step was to evaluate the extent of the risk conferred by rs850678541 and rs851590509 in our UK Golden Retriever set of 37 MCT cases and 53 controls. TaqMan genotyping of SNP rs851590509 in this set showed that the alternative ‘*A*’ allele is extremely common (83% of the dogs, including 74% of controls, carried at least one copy, and 42% of the dogs, including 21% of controls, carried two copies), and has a statistically significant association with MCTs (P-value = 1.5 x 10^−7^) (Table 5). Furthermore, a combined analysis of rs850678541 and rs851590509 in this set of Golden Retrievers demonstrated a statistically significant association with MCTs (P-value = 2.6 x 10^−8^) and revealed that collectively these variants explain 29% of the MCT trait in this breed (Table 5). Due to the rarity of the rs851590509 SNP in the Labrador Retriever set we could not perform a combined analysis of rs851590509 and rs850678541 in this breed.

## Discussion

In this study we have identified a synonymous germline variant (‘*A*’ allele of SNP rs850678541) in the *DSCAM* gene that is associated with the elevated risk of MCT development in Labrador Retrievers. We revealed that, although the variant has no effect on *DSCAM* mRNA expression, it is associated with a significantly reduced DSCAM protein level in MCTs and in normal skin. The demonstration that intronic variants at loci in the *DSCAM* gene that are in LD with SNP rs850678541 do not cause alternative exon splicing (that may be reflected in a decrease in the level of the full length 196kDa DSCAM protein—UniProtKB F1PA86_CANLF) affords a strong indication that the SNP rs850678541 alternative allele may be responsible for the significant reduction in DSCAM protein expression observed in MCTs and normal skin specimens from Labrador Retrievers homozygous for the alternative allele. The variant allele is common in Labrador Retrievers, is associated with a per allele increase in MCT risk of 1.66 x, and is estimated to account for 2% of the MCT trait in the breed.

SNP rs850678541 was also shown to be a risk factor for MCT development in Golden Retrievers (accounting for 5% of the MCT trait in the breed), suggesting that the variant arose in a common ancestor at some point prior to divergence of the Labrador and Golden Retriever breeds. The strength of the association (odds ratio = 2.11) between the SNP and MCTs in our set of Golden Retrievers suggests that a lack of statistical power may be the reason why an association to SNPs in the vicinity of CFA31 34.7Mb was not detected in the European Golden Retriever MCT GWAS performed by Arendt and colleagues [22]. An alternative explanation for this is that the CFA31 SNPs on the canineHD array were not able to ‘capture’ SNP rs850678541 in Golden Retrievers due to a different haplotype structure in this breed. The association between the CFA20 SNP rs851590509 and MCTs in European Golden Retrievers reported by Arendt and co-workers [22] was reproduced in our set of Golden Retrievers. Significantly, our combined analysis showed that collectively SNPs rs850678541 and rs851590509 explain 29% of the MCT trait in Golden Retrievers. In our set of Labrador Retrievers, SNP rs851590509 was very rare, which did not allow for a combined analysis to be undertaken. To the best of our knowledge, the SNP rs850678541 described here is currently the only MCT-associated variant in Labrador Retrievers to be identified, although it is likely that other MCT-associated variants will be described because our secondary GWAS meta-analysis has suggested associations with other genomic regions. Furthermore, the demonstration that MCT susceptibility loci are shared by Labrador and Golden Retrievers, suggests that meta-analysis of genotype data from both breeds may uncover additional MCT susceptibility loci.

A recent study demonstrated the presence of Mendelian disease variants in pedigree dog breeds for which the disease/an elevated risk of developing the disease had not previously been reported [36], leading the investigators to speculate that the ‘genetic background’ may affect how a mutation is manifest. The most notable example is arguably the *SOD1*:*c*.*118A* allele, homozygotes and heterozygotes of which in 5 breeds are associated with degenerative myelopathy. The *SOD1*:*c*.*118A* allele is also present (at up to a high frequency) in many breeds [37] that are not known to develop degenerative myelopathy, suggesting that the penetrance of the allele is affected by other genetic or environmental factors. In this study we found that the SNP rs850678541 alternative ‘*A*’ allele, which is associated with MCTs in Labrador and Golden Retrievers, is present at a lower frequency in Border Collies (frequency 12.3 x lower) and Cavalier King Charles Spaniels (frequency 1.3 x lower), two breeds that are under-represented amongst MCT-affected dogs [4, 19]. As MCT susceptibility appears to be complex, the risk conferred by the SNP rs850678541 alternative ‘*A*’ allele in Labrador and Golden Retrievers has to be considered in the context of potential modifying alleles at other MCT susceptibility loci that may be present in Labrador and Golden Retrievers and absent from other breeds. Indeed, susceptibility variants have been found to modify the risk of breast cancer development associated with the *BRCA1* and *BRCA2* mutations, thereby accounting for the variation in breast cancer penetrance observed for these mutations in different human families [38]. Extensive GWAS of human diseases has demonstrated that genetic risk factors underlying complex diseases, such as cancer, comprise both common ancestral risk variants of intermediate effect and rarer risk variants of higher effect/penetrance [39]. However, it is likely that in the dog, as is the case for diverse human populations, the impact of these risk variants will depend on both environmental influences and other genetic risk factors that an individual possesses. In this study we have identified a common risk variant of intermediate effect that we have shown to be reproducibly associated with MCTs in two breeds. This suggests that the common disease common variant hypothesis for human complex disease also holds true in the dog, although this may vary between breeds. It will ultimately be informative to genotype all subsequently identified Labrador and Golden Retriever MCT susceptibility variants in low risk breeds to assist understanding of the contribution of the ‘interaction’ between susceptibility loci to the elevated risk of MCT development.

For some time, synonymous variants, such as SNP rs850678541, were known as silent, as it was thought that they had no effect on gene expression and cellular fitness. Genome sequencing led to the realisation that synonymous codons do not appear with the same frequency in a genome (a phenomenon known as codon usage bias) and challenged this concept [30]. Consequently, it is now acknowledged that synonymous variants can influence cellular functions through effects on mRNA stability and processing, translation kinetics and protein folding [40]. Interestingly, Vedula and co-workers have shown that the diverse functions of β and ϒ actin homologues are defined by synonymous variants in their nucleotide sequences, and consequent differences in their translation and post-translational modifications dynamics, demonstrating that synonymous variants are important factors in the regulation of the functional diversity of protein isoforms in a variety of physiological conditions [41]. With regards to medical conditions, synonymous mutations have been associated with complex diseases such as neurological disorders, diabetes and cancer [42]. In a study in which 3,000 tumour exomes and 300 tumour genomes were analysed it was estimated that 1 in 5–1 in 2 silent mutations were positively selected, and acted as driver mutations in human cancers [43]. With regard to canine MCTs, the Golden Retriever MCT-associated variant SNP rs851590509 identified by Arendt and co-workers is also of a synonymous nature [22]. In this case, the synonymous variant is located in a splicing site, and was shown to have an effect on splicing [22]. By contrast, in the present study the synonymous SNP that we have shown to be associated with MCTs in Labrador and Golden Retrievers (SNP rs850678541) appears to have an effect on the translation dynamics of the *DSCAM* gene.

Translation dynamics are affected by the decoding times of each of the codons present in a transcript [44]. The decoding time of each codon is a function of parameters such as the overall codon landscape in the transcript, and is also positively correlated with abundance of the cognate tRNA [45]. Transfer RNA abundance varies between different tissues [46], and is positively correlated with the frequency with which the codon that is cognate to a tRNA is used in genes that are ‘highly expressed’ in a given tissue [47]. Therefore, a synonymous mutation can conceivably lead to an increased decoding time and impaired translation of a transcript in a given tissue if it results in a rarer codon than the ‘wild type’. Indeed, Kirchner and co-workers identified a synonymous SNP in the cystic fibrosis transmembrane conductance regulator gene, which resulted in a rare codon, which had a low-frequency cognate tRNA, and decreased protein expression in bronchial tissue. Remarkably, they showed that increasing the abundance of the tRNA cognate to the mutated codon rescued the protein expression phenotype associated with the synonymous SNP [48]. We were unable to measure, in our MCT biopsies and skin specimens, the abundance of the tRNAs cognate to the ‘reference’ (*CGC*) codon and alternative (*CGU*) arginine codon generated by the synonymous variant. This is because tRNA microarrays are unable to differentiate between these two arginine isoacceptors, and the partial hydrolysis which is used to overcome the challenges imposed by tRNA secondary and tertiary structures to build a next generation sequencing library, makes it impossible to differentiate (and quantify the relative abundances of) the tRNAs by sequencing [49]. Furthermore, a sequence (canine or human) for the arginine ‘reference codon’ cognate tRNA is not available in the tRNA database [50], which made the design of primers for a RT-qPCR assay impossible. Therefore, unfortunately, we were unable to mechanistically correlate the reduced levels of the DSCAM protein that we observed with the synonymous SNP identified, although we are hopeful that future advances in tRNA analysis techniques will enable us to so do. Nevertheless, the fact that the ‘reference’ CGC codon is nearly three times as frequent as the rs850678541 alternative allele-containing *CGU* codon in a sample of 1,194 canine mRNA transcripts (Kazusa database [51]; S7 Table) is an indication that the synonymous variant that we identified might be capable of having a negative effect on the *DSCAM* gene translation dynamics. Interestingly, 10-fold differences between the translation efficiencies of arginine codons have been demonstrated in plant chloroplasts where there was parity in codon usage [52].

The *DSCAM* gene was first characterised as encoding a cell adhesion molecule; a member of the immunoglobulin superfamily of cell surface proteins, in a study which identified it as a Down syndrome-related gene [53]. It has an important function in nervous system development, and its conservation in arthropods and mammals reflects its role in neural circuitry formation and an innate-immunity function, specific to arthropods [54, 55]. *DSCAM* has also been identified as a predisposing locus for Hirschsprung’s disease that is often observed in association with Down syndrome [56]. SNPs in the *DSCAM* gene have also been associated with idiopathic scoliosis in adolescents [57] and with anxiety and depression disorder [58]. Although a germline SNP in the *DSCAM* gene has been found to be associated with shortened overall survival in response to chemotherapy in patients with non-small cell lung cancer [59], and somatic mutations in this gene have been found in approximately 40 different types of tumour ([60]; S8 Table), to the best of our knowledge, this is the first report of an association between a germline variant in the *DSCAM* gene and susceptibility to cancer.

It is likely to be significant that the development of MCTs in two susceptible canine breeds has now been associated with germline variants in genes involved in cell-to-cell or cell-to-extracellular matrix (ECM) interactions. MCT development has been associated with a variant (SNP rs851590509) in the gene of a G-protein subunit (*GNAI2*), which acts as regulator of different transmembrane signalling pathways in a study of European Golden Retrievers; and with variants located in a region containing genes that encode hyaluronidase, an enzyme which cleaves a component of the MCT ECM, in a study of US Golden Retrievers [22]. Strikingly, in a study of US Labrador Retrievers, MCT development was found to be associated with a variant suspected to be located in a gene encoding a subunit of integrin, a cell adhesion and signalling molecule [23]. Our finding that MCT development is associated with a variant in the *DSCAM* gene in European pet Labrador and Golden Retrievers is additional evidence that alterations in the interaction of mast cells with the microenvironment is an important step in MCT tumorigenesis. More specifically, in the case of Labrador Retrievers, it provides compelling evidence that alterations in cell adhesion molecules represent an important risk factor for MCT development. Indeed, it has been shown that cell adhesion molecules, such as E-Cadherin, and the Ig superfamily member CADM1, can act as tumour suppressors mainly through contact inhibition of cell proliferation [61–64]. For dogs affected by MCTs, there was a trend for those whose MCTs displayed a reduced expression level of SynCAM, a cell adhesion molecule of the immunoglobulin superfamily, to be more likely to suffer MCT-related death [65]. In this study we found no association between SNP rs850678541 alternative allele and MCT metastasis in Labrador Retrievers. However, the sample set was of limited size and the thirteen dogs for whom definitive confirmation of ‘MCT metastatic disease status’ was achievable did not include dogs homozygous for the SNP rs850678541 alternative (variant) allele, a significant exclusion given that the ten-fold reduction in the level of DSCAM protein expression was only observed in MCTs and skin biopsies from dogs which are homozygous for the SNP rs850678541 alternative allele. Consequently, a much larger investigation featuring dogs with all three SNP rs850678541 genotypes, and affected by both metastasising and non-metastasising MCTs, is merited. Illustration of the likely importance of dysregulation of cell adhesion in human mastocytosis is the fact that the pathway activated by the *c-kit* receptor, which is frequently found somatically mutated in human mastocytosis, also regulates mast cell adhesion, in addition to survival and other cellular processes [66, 67].

In conclusion, the results presented here demonstrate the importance of retaining synonymous variants as possible functional candidates when screening for germline susceptibility loci for complex diseases, such as cancer. In addition, through identifying a common genetic risk factor for MCT development in Labrador and Golden Retrievers, the contribution of dysregulation of cell adhesion to MCT pathogenesis has been demonstrated.

## Methods

### Ethics statement

The blood samples and buccal swabs used in the study were collected, retained and used for research with the written consent of the dogs’ owners. Buccal swabs were collected by dogs’ owners, and blood samples were collected by clinicians with the consent of dogs’ owners. Blood samples from UK dogs were surplus to that collected for a clinical reason, or as part of a health check. MCT biopsies were dissected (with the consent of dogs’ owners) from MCTs which were surgically removed in the course of standard treatment protocols. Biopsies of normal skin were excised post-mortem from dogs whose bodies had been donated for research by their owners. The research study, and the protocol by which samples were collected for the study, were approved by the ethics committees of the participating institutions: AHT Clinical Ethics Committee, project number AHT_07–11; Committee for Animal Care at the Massachusetts Institute of Technology, approval number MIT CAC 0910-074-13; Uppsala Animal Ethical Board, approval number C2-12; Animal Experiments Committee of the Academic Biomedical Centre, Utrecht, The Netherlands, experimental protocol ID 2007.111.08.110.

### Germline DNA samples

Buccal swabs and blood samples were collected from Labrador and Golden Retrievers confirmed by histopathology to have/have had a MCT, and Labrador and Golden Retrievers aged at least 7 years old whom had never been affected by any form of cancer. For GWAS, Labrador Retriever samples were collected by the Animal Health Trust in the UK (153 samples), the Broad Institute in the United States (108 samples), and the University of Utrecht in the Netherlands (77 samples) (S9 Table). For genotyping of candidate germline MCT susceptibility variants, 407 Labrador Retriever samples were collected by the Animal Health Trust in the UK (S9 Table). All Golden Retriever, Border Collie and Cavalier King Charles Spaniel samples were collected by the Animal Health Trust in the UK. Genomic DNA was isolated from buccal swabs by phenol-chloroform extraction [68], and from whole blood using the Nucleon Genomic DNA Extraction Kit (Tepnel Life Sciences), or the QIAamp DNA Blood Midi Kit (Qiagen).

### DNA, RNA and protein extraction from RNAlater-preserved tissue biopsies

This protocol is available on the protocols.io database (dx.doi.org/10.17504/protocols.io.sq2edye). Seventeen RNAlater (ThermoFisher Scientific)-preserved MCT (Biopsies#1–17 in Fig 2) and three post-mortem normal skin biopsies (Biopsies#18–20 in Fig 2), in the form of 3mm cubes, were homogenised in 700μl of Qiazol (Qiagen) by shaking with 2 x 7mm stainless steel beads at 30Hz in a TissueLyser LT (Qiagen) for 10 min at room temperature. Chloroform (140μl) was added to each homogenate and the aqueous phase recovered following centrifugation (12,000 x g for 15 min at 4°C) was used for RNA extraction with the miRNeasy Mini Kit (Qiagen), following the manufacturer’s instructions.

The interphase and organic phase were used for DNA and protein extraction. Briefly, DNA was precipitated with 100% (v/v) ethanol, and washed successively in 0.1M sodium citrate and 75% (v/v) ethanol before being resuspended in 8mM sodium hydroxide. Following DNA precipitation, protein was precipitated from the interphase and organic phase with 100% (v/v) isopropanol, washed successively in 0.3M guanidine-hydrochloride in 95% ethanol, and 75% (v/v) ethanol, and resuspended in 10M urea, 1% (v/v) 2-mercaptoethanol.

### Genome wide association analysis (GWAS)

Genotyping was performed at the Centre National De Genotypage, Paris, France. Genomic DNA (200ng at 100ng/μl) was genotyped using the Infinium HD Ultra Assay (Illumina) and the canineHD array (Illumina), which comprises 173,662 SNPs spanning the canine genome at a density of around 70 SNPs per Mb [69]. GWAS datasets were analysed individually by country and genotyping run before meta-analysis to preserve data quality and reduce possible biases caused by different sample preparation procedures in different laboratories, and possible population effects between countries (case-control sets did not all approximate to a 1:1 ratio). The number of cases and controls in each individual dataset following sample quality control (QC) filtering (dropping individuals with a SNP call rate of < 90%) are shown in S1 Table. SNP QC filtering was conducted in each of the individual datasets independently. SNPs that had a minor allele frequency (MAF) of <5% and/or call rate of <97% in each dataset were excluded.

Within each dataset we visually assessed the extent of population substructure using multidimensional scaling plots in two dimensions, and by calculating genomic inflation factors, which were estimated for each dataset independently from the median of the Χ^2^ tests of all SNPs tested following QC (S1 Table). From examination of the multidimensional scaling plot for the “Set 1” dataset it was apparent that there were two distinct clusters of dogs within this dataset there were 28 MCT cases and 20 controls that were Guiding Eye for the Blind Dogs (S6 Fig). As these dogs originate from a line of Labradors distinct from the general pet population we postulated that they could be potential confounders in the GWAS analyses. We therefore excluded Guiding Eye for the Blind Dogs from this dataset and from future analyses. Unadjusted GWAS analyses were conducted using PLINK [70] and analyses correcting for population stratification were performed using GEMMA [71].

Genome-wide meta-analyses were conducted using SNPs that had passed QC within two or more individual datasets (S2 Fig), and for the population-adjusted meta-analysis (S3 Fig) using only SNPs in common in all six datasets.

### Sequence capture, next generation sequencing, and variant identification

Genomic regions implicated by GWAS as containing MCT susceptibility loci were captured from DNA samples from affected and unaffected Labrador Retrievers using SureSelect Target Enrichment System RNA oligonucleotide baits (Agilent) from libraries prepared using the TruSeq DNA Sample Preparation Kit (Illumina index set A; Illumina). Enriched libraries were sequenced using a HiSeq 2000 (100bp paired-end sequencing, approximately 30-fold coverage) (Illumina). A Genome Analysis Toolkit (GATK)-based pipeline [72] was employed to align Fastq file format sequence reads to the CanFam3.1 reference Boxer genome and detect SNVs and indels. The potential functional impact of each variant was predicted using Variant Effect Predictor [73] and SIFT [25]. A locus harbouring one or more allelic variants was considered to be a candidate MCT susceptibility locus, and selected for further analysis, if it fulfilled both of the following criteria:

1. Locus position: exon, including UTRs, and predicted to be deleterious or non-deleterious, OR splice region
                                                                  AND
2. Segregation: One allele is present as at least one copy in at least one case and is not present in any of the controls [i.e. (a) Biallelelic loci: one allele can be present in both cases and controls, but the second allele must be unique to the cases; (b) Multi-allele loci: multiple alleles can be present in both cases and controls, but one allele must be unique to the cases)

### Genotyping of candidate MCT susceptibility variants

All CFA31 candidate MCT susceptibility variants were typed in Labrador Retrievers, and SNP rs850678541 was typed in Golden Retrievers, Border Collies and Cavalier King Charles Spaniels. For SNPs, TaqMan Genotyping Assays (ThermoFisher Scientific) were designed (S8 Table) from variant-containing genomic DNA sequences in which known SNPs, repeat sequences, and stretches of sequence displaying significant similarity to other regions of the genome were masked. TaqMan Genotyping Assays were 10μl reactions performed using 1μl of genomic DNA, according to the manufacturer’s instructions. The TaqMan Genotyping Master Mix (ThermoFisher Scientific) was used routinely, but the TaqPath ProAmp Master Mix (ThermoFisher Scientific) was employed when there was an indication of PCR inhibition. Thermocycling was performed in a StepOne Plus Machine (ThermoFisher Scientific), and the results analysed using TaqMan Genotyper Software (ThermoFisher Scientific). Every genotyping run featured DNA samples of known genotype as positive controls, and two non-template negative controls.

The indel variant at CFA31:34667505 was genotyped through DNA fragment analysis. Amplification and fluorescent end-labelling of target fragments was achieved using 10μl PCR reactions containing 2μl of 1:100 diluted genomic DNA sample, 0.2μM of each of a forward FAM-labelled forward primer and an unlabelled reverse primer (S9 Table), 4 x 0.2mM dNTPs and 0.25 units of HotStarTaq DNA Polymerase (Qiagen). Thermocycling was performed in a T100 Thermal Cycler (BioRad) using the following parameters: 95°C, 15 min; (94°C, 30s; 60°C, 60s; 72°C, 30s) x 35; 72°C, 10 min. A microlitre of each labelled PCR product was mixed with 10μl of HiDi formamide (ThermoFisher Scientific) and 0.4μl of the ABI GeneScan 400HD ROX size standard (ThermoFisher Scientific) and loaded into an ABI 3130xl Genetic Analyser machine, using POP_7 polymer (ThermoFisher Scientific) as the separation matrix. The resulting data were analysed using the ABI GeneMapper software (ThermoFisher Scientific). Every genotyping run featured positive and negative controls.

### Genotyping of CFA20 SNP rs851590509 in Labrador and Golden Retrievers

The Golden Retriever MCT-associated SNP rs851590509, identified by Arendt and colleagues [22], was genotyped in a set of Labrador and Golden Retrievers by TaqMan assay (Thermofisher Scientific) (S8 Table). Genotyping was performed using 10μl reactions, incorporating 1μl of genomic DNA and the TaqPath ProAmp Master Mix (ThermoFisher Scientific), according to the manufacturer’s instructions. Thermocycling was performed in a StepOne Plus Machine (ThermoFisher Scientific), and the results analysed using TaqMan Genotyper Software (ThermoFisher Scientific). Every genotyping run featured two non-template negative controls, and a positive control (a sample of known genotype).

### Linkage Disequilibrium (LD) analysis

Variant-harbouring loci in LD with the SNP rs850678541 were identified using genotypes derived from the resequencing data obtained for 12 Labrador Retrievers (six cases and six controls). Haplotype analysis of biallelic loci was performed using Haploview, version 4.2 [74]. The software’s “Tagger” function [75], with a r^2^ threshold of 0.8, was used to identify biallelic variants in LD with the rs850678541 variant. The identification of multiallelic loci in LD with SNP rs850678541 was performed in two steps. The first step involved the identification of loci for which one allele had a frequency = the frequency of the SNP rs850678541 alternative (variant) allele ± 20%. In the second step, the genotypes at the loci selected in step one were compared to the genotype of the SNP rs850678541 locus, in order to identify those that displayed ≤1 segregation event from this locus.

### Reverse transcription-quantitative PCR (RT-qPCR)

MCT RNAs with RIN values ≥8.0 (Agilent Bioanalyser RNA 6000 Nano Kit; Agilent) were treated with 1.5U/μg RNA of heparinase I (Sigma-Aldrich) in 5mM Tris-HCl (pH 7.5), 1mM CaCl_2_ at 25°C for 3h in order to eliminate heparin, a reverse transcription and PCR inhibitor commonly found in mast cells [31]. cDNA was prepared from 2.44μg of heparinase- treated RNA, using the High Capacity RNA to cDNA kit (ThermoFisher Scientific), following the manufacturer’s instructions. Each MCT cDNA sample was assessed for the presence of PCR (and potentially reverse transcription) inhibitors by adding an equal amount of a synthetic *Solanum tuberosum*-derived amplicon to each sample, and screening for differences between the synthetic amplicon quantification cycle (Cq) value obtained for each cDNA sample upon PCR amplification [76]. PCR reactions (10μl), comprising 1μl of cDNA, 1 x SsoAdvanced SYBR Green Master Mix (BioRad), 1.33fM of SPUD amplicon (S9 Table), and 0.3μM of forward and reverse SPUD primers (S9 Table), were run in an ABI StepOne Plus machine (ThermoFisher Scientific) using the following program: 98°C, 2min; (98°C, 5s; 60°C, 30s) x 40; Melt Curve program. Triplicate PCR assays were performed for each MCT cDNA sample and a mean Cq value calculated.

*DSCAM* mRNA expression was assayed using 10μl PCR reactions, comprising 1μl of cDNA, 1 x PowerUp SYBR Green Master Mix (ThermoFisher Scientific) and 0.3μM of forward and reverse DSCAM primers (S9 Table), run in an ABI StepOne Plus machine (ThermoFisher Scientific) with the following parameters: 50°C, 2 min; 95°C, 2 min; (95°C, 3s; 60°C, 30s) x 40; Melt Curve program. Triplicate PCR assays were performed for each MCT cDNA sample. To enable normalisation of *DSCAM* expression values, the expression level of a 70bp fragment of a SINE [77] that occurs in the 3’-untranslated region of hundreds of canine mRNAs, in each MCT RNA sample was also assayed (performing triplicate reactions for each cDNA sample). A repeat sequence that is present in hundreds of copies in any canine tissue sample transcriptome will effectively display invariant expression across all samples of a given tissue type ensuring reliable normalisation of RT-qPCR-derived gene expression data [78]. The SINE PCR reaction master mix was subject to UV irradiation (302nm) for 5 min prior to the addition of the SINE PCR primers (S9 Table), but the PCR reaction components and thermocycling parameters were as used for the *DSCAM* mRNA assays. For each MCT cDNA sample, a mean Cq value was determined for each PCR amplicon from the Cq values obtained for the triplicate PCR reactions by the StepOne Plus software (Thermofisher Scientific). The mean *DSCAM* Cq value for each MCT cDNA sample was imported into qbase^+^ (Biogazelle), which generated a relative measure of *DSCAM* expression (a calibrated normalised relative quantity) for each MCT cDNA sample, using the mean SINE Cq value obtained for the same cDNA sample [79].

### End point PCR assays

Nested end point PCR assays were performed to screen for possible alternative splicing of *DSCAM* mRNAs. For the E14-15 Assay, first round PCR reactions (20μl) featured 1μl of cDNA, 1 x HotStar HiFidelity PCR buffer (Qiagen), 1μM of both the ‘external’ (A) forward and reverse ‘external’ (A) primers (S9 Table), and 1 unit of HotStar Taq HiFidelity DNA Polymerase (Qiagen). Thermocycling was performed as follows: 95°C, 5 min; (94°C, 15s; 52°C, 60s; 72°C, 1 min) x 40; 72°C, 10 min,. A 1μl aliquot of a 1 : 100 dilution of each first round PCR product was used in a 20μl second round PCR reaction comprising 1 x HotStar HiFidelity PCR buffer (Qiagen), 1μM of both the ‘internal’ (B) forward and reverse primers (S9 Table), and 1 unit of HotStar Taq HiFidelity DNA Polymerase (Qiagen). The thermocycling parameters were 95°C, 5 min; (94°C, 15s; 51°C, 60s; 72°C, 60s) x 30; 72°C, 10 min. For the E15-17 Assay, first round PCR reactions (20μl) featured 1μl of cDNA, 1 x HotStar Taq PCR buffer (Qiagen), 1 x Q-Solution (Qiagen), 0.5μM of both the ‘external’ (A) forward and reverse primers (S9 Table), 0.3mM of each of 4 x dNTPs (Qiagen), 2 units of HotStar Taq DNA Polymerase (Qiagen), and 0.08 units of HotStar HiFidelity Taq DNA Polymerase (Qiagen). Thermocycling was performed as follows: 95°C, 2 min; (94°C, 10s; 56°C, 60s; 68°C, 6 min 30s) x 40. A 1μl aliquot of a 1 : 100 dilution of each first round PCR product was used in a 20μl second round PCR reaction comprising 1 x HotStar Taq PCR buffer (Qiagen), 1 x Q-Solution (Qiagen), 0.5μM of both the ‘internal’ (B) forward and reverse primers (S9 Table), 0.3mM of each of 4 x dNTPs (Qiagen), 2 units of HotStar Taq DNA Polymerase (Qiagen), and 0.08 units of HotStar HiFidelity Taq DNA Polymerase (Qiagen). Thermocycling was performed as follows: 95°C, 2 min; (94°C, 10s; 59°C, 60s; 68°C, 6 min 30s) x 40. Second round PCR reaction products were analysed by 2% agarose gel electrophoresis (E14-15 Assay) and by 0.8% agarose gel electrophoresis (E15-17 Assay), respectively. Images were captured using the Alpha Imager (Alpha Innotech).

### Semi-quantitative western blot

Protein samples were quantified using the Bradford Assay. Prior to polyacrylamide gel electrophoresis, protein samples were mixed with 4 x NuPage loading buffer (ThermoFisher Scientific), incubated at 70°C for 10 min, and on ice for 5 min. Twenty-five micrograms of each protein sample and 10μl of the Precision Plus Western C protein standard (BioRad) were loaded onto a TGX Stain-Free 4–20% gradient gel (BioRad) and electrophoresed at 200kV for 40 min in 1 x Tris-Glycine SDS PAGE Buffer (National Diagnostics). A single protein sample was included on every gel for use as an inter-western blot calibrator. Prior to transfer of proteins to a membrane, a gel was exposed to 365nm UV for 2.5–5 min in order to activate the Stain-Free technology. Proteins were transferred from the gel to a 0.45μm nitrocellulose membrane (BioRad) in a Mini Trans-Blot Cell system, at 100kV for 1 hour in Tris-Glycine transfer buffer, containing 20% (v/v) methanol.

The Stain-Free total protein image of a protein blot was detected under UV light using the Alpha Imager (Alpha Innotech). The membrane was subsequently agitated in Ponceau S solution (Sigma Aldrich) for 1 min, washed 3 x with MilliQ water and visualised under white reflective light in the Alpha Imager (Alpha Innotech). Ponceau S stain was removed by 3 x washes in MilliQ water, and a membrane gently agitated in Blocking Solution (WesternBreeze Chromogenic Western Blot Immunodetection Kit; ThermoFisher Scientific) for 30 min, and incubated in a 1: 1000 dilution of anti-DSCAM antibody (abcamab85362, which has a highly conserved human DSCAM protein sequence as epitope) in Blocking solution at 4°C overnight. The membrane was washed 3 x (5 min each) with the Antibody Wash Solution (WesternBreeze Chromogenic Western Blot Immunodetection Kit; ThermoFisher Scientific), incubated with a 1 : 1000 dilution of alkaline phosphatase-conjugated Goat anti-Rabbit IgG (H+L) secondary antibody (ThermoFisher Scientific) in Blocking Solution for 30 min, washed 3 x (5 min each) with the Antibody Wash Solution, and finally incubated with BCIP/NBT Chromogenic substrate (WesternBreeze Chromogenic Western Blot Immunodetection Kit; ThermoFisher Scientific) for 1–5 min. Images were captured, under reflective white light, on the Alpha Imager (Alpha Innotech). The total protein and DSCAM staining membrane images were imported into the ImageLab software (BioRad) for analysis and quantification. Normalisation of the DSCAM level in each sample involved reference to the total quantity of protein detected (by Ponceau S or the Stain-Free technology) in the sample, and inter-membrane calibration using the ratio of DSCAM protein quantity/total protein quantity measured for the 25μg protein sample loaded onto every gel.

### Statistical analysis

#### GWAS

Analyses were performed using STATA 10.0 (College Station, TX, USA) using a fixed effects model and inverse-variance weighted averages of either the logarithm of the odds ratios from PLINK and their standard errors (population-unadjusted meta-analyses, Fig 1 and S1 Fig), or of beta coefficients and standard errors using GEMMA (S3 Fig). Heterogeneity was assessed using the Q statistic.

#### Association analysis between MCTs (Labrador and Golden Retrievers) and candidate MCT susceptibility variants

Association analyses were conducted in STATA 10.0. SNPs were analysed using logistic regression and log likelihood ratio tests using a linear per allele model. Indel association analysis was performed using the Fisher’s exact test using data coded in both a genotypic and allelic form. The association between MCT and SNP rs851590509 in the Labrador Retriever was tested using the Fisher’s exact test due to the rarity of the variant in this breed. For the Golden Retriever, logistic regression and log likelihood ratio tests were used to test a statistical model comprising SNPs rs851590509 and rs850678541 with MCT to provide an overall odds ratio, 95% confidence interval and P-value. Logistic regression was used to compute McFadden’s pseudo r^2^; i.e. goodness of model fit for tested variants, and a model containing both SNPs rs851590509 and rs850678541 combined.

#### Quantitative analysis of DSCAM mRNA expression by RT-qPCR

Statistical analysis featured comparisons between the relative measures of *DSCAM* mRNA expression (SINE-normalised *DSCAM* mRNA expression value) of individual MCT biopsies belonging to the three genotype groups: (a) homozygous for the SNP rs850678541 reference ‘G’ allele, (b) homozygous for the SNP rs850678541 alternative ‘A’ allele, and (c) heterozygous. Applying non-parametric tests on the assumption of non-normal distributions of relative *DSCAM* mRNA expression values, the Kruskal-Wallis test was employed to compare *DSCAM* mRNA expression between the three genotype groups, and the Mann-Whitney U test (two-tailed) employed to compare *DSCAM* mRNA expression between pairwise combinations of the three genotype groups.

#### Quantitative analysis of DSCAM protein expression by semi-quantitative western blot

Statistical analysis featured comparisons between the relative measures of DSCAM protein expression [DSCAM protein expression value normalised using a) total quantity of the protein sample concerned loaded onto a gel, and then b) inter-membrane calibrator of individual MCT biopsies] belonging to the three genotype groups: (a) homozygous for the SNP rs850678541 reference ‘G’ allele, (b) homozygous for the SNP rs850678541 alternative ‘A’ allele, and (c) heterozygous. Applying non-parametric tests on the assumption of non-normal distributions of relative DSCAM protein expression values, the Kruskal-Wallis test was employed to compare DSCAM protein expression between the three genotype groups, and the Mann-Whitney U test (two-tailed) employed to compare DSCAM protein expression between pairwise combinations of the three genotype groups.

## Supporting information

S1 Table The numbers of cases and controls, and genomic inflation factors, for individual GWAS datasets. ^#^Number of cases and controls and genomic inflation factors for GWAS dataset before exclusion of Guiding Eye for the Blind Dogs. *Number of control dogs before exclusion, in the meta-analysis including Sets 1–6, of one individual that subsequent to genotyping had been reported as being affected by cancer (not MCT).
(TIF)

S2 Table The number of DNA samples from Labrador Retrievers with a MCT or without a MCT (Control) used for candidate MCT susceptibility loci genotyping. The percentage of DNA samples that were successfully genotyped by each assay is indicated.
(TIF)

S3 Table The results obtained for the SPUD assay for detection of reverse transcription and/or PCR inhibitors in MCT biopsy RNA samples. SNP rs850678541 genotypes are represented by: *ALT/ALT*—Alternative (variant) ‘A’ allele homozygote; *REF/REF*—Reference ‘G’ allele homozygote; *REF/ALT*—GA heterozygote.
(TIF)

S4 Table Raw quantitative RT-PCR data. (TIF)

S5 Table Raw western blot data. (TIF)

S6 Table The 19 x *DSCAM* biallelic variants and three multiallelic variants shown to be in LD with SNP rs850678541. The table containing the biallelic variants shows the r^2^ values obtained from analysis performed using the “Tagger” function of Haploview, using a r^2^ threshold of 0.8 and SNP rs850678541 as a tagger. The variants’ locations in the *DSCAM* gene are also listed. Intron 14–15—the variant is located in the intron between exons 14 and 15; Intron 15–16—the variant is located in the intron between exons 15 and 16.
(TIF)

S7 Table Codon usage information for the Arginine (R) synonymous codon family, extracted from the Kazusa database of codon usages [51], based on 1,194 canine transcripts. Information pertaining to the reference *CGC* codon is highlighted in green, and to the alternative *CGT* codon is highlighted in yellow.
(TIF)

S8 Table Tissue distribution of somatic mutations in the *DSCAM* gene found in human cancers. Source: COSMIC database [60].
(TIF)

S9 Table Labrador Retriever sample sets used in the GWAS and candidate MCT susceptibility variant TaqMan genotyping. M: Male; F: Female; (N): neutered; NA: Not available; N/A: Not applicable.
(TIF)

S10 Table Probes and PCR primers used in custom TaqMan genotyping assays. (TIF)

S11 Table PCR assay reagents used for indel genotyping, screening for reverse transcription/PCR inhibitors, and assay of *DSCAM* expression and alternative splicing. (TIF)

S1 Fig QQ plots for GWAS meta-analysis of three Labrador Retriever datasets (Sets 1–3). Red spots denote chi-squared values expected under the null for each of the number of SNPs tested; blue spots denote the observed chi-squared values for each SNP.
(TIF)

S2 Fig GWAS meta-analysis of MCT in the Labrador Retriever. **A.** Manhattan plot of a combined analysis of 173 cases and 112 controls from six case-control sets. Analyses comprised 118,628 SNPs. The horizontal red line denotes the genome-wide association threshold based on Bonferroni correction for 118,628 tests (P-value = 4.2 x 10^−7^). The plot was generated using Haploview version 4.2 [74]. **B.** QQ plot for GWAS meta-analysis of six Labrador Retriever datasets. Red spots denote chi-squared values expected under the null for each of the number of SNPs tested; blue spots denote the observed chi-squared values for each SNP.
(TIF)

S3 Fig GWAS meta-analysis of MCT in the Labrador Retriever adjusted for population stratification. **A.** Manhattan plot of a combined analysis of 173 cases and 112 controls from six case-control sets. Analyses comprised 87,632 SNPs. The horizontal red line denotes the genome-wide association threshold based on Bonferroni correction for 87,632 tests (P-value = 5.7 x 10^−7^). The plot was generated using Haploview version 4.2 [74]. **B.** QQ plot for GWAS meta-analysis of six Labrador Retriever datasets after adjustment for population stratification. Red spots denote chi-squared values expected under the null for each of the number of SNPs tested; blue spots denote the observed chi-squared values for each SNP.
(TIF)

S4 Fig Total protein images (obtained through either Stain-Free technology or Ponceau staining) of protein samples obtained from Labrador Retriever MCT biopsies. MCT biopsies #2, 3, 9 and 11 were not utilised for assay of DSCAM protein expression because the presence of an intensely staining ~15kDa band in each protein sample suggested significant protein degradation. The ~15kda band was significantly ‘weaker’ in the MCT biopsy #6 protein sample that was employed as an inter-membrane calibrator (for normalisation of DSCAM protein levels).
(TIF)

S5 Fig **A. Whole western blot images of DSCAM antibody staining and total protein staining (through Ponceau staining and Stain-Free technology) of protein samples extracted from Labrador Retriever MCT biopsies (#1–17) and normal skin biopsies (#18–20).** Sample number colours indicate SNP rs850678541 genotype: Red = *Alt/Alt* [Alternative (variant) ‘*A*’ allele homozygote]; Green = *Ref/Alt [G/A* heterozygote]; Blue = *Ref/Ref* [Reference ‘*G*’ allele homozygote]. **B.** Bar charts showing the DSCAM level in each MCT biopsy normalised by the total quantity of the MCT biopsy protein (assayed by the Stain-Free technology) present on the membrane. The biopsies are grouped according to their SNP rs850678541 genotype. *P≤0.05 (Mann-Whitney U test). **C.** Bar charts showing the mean DSCAM protein level +/- SD of each MCT biopsy SNP rs850678541 genotype group. Error bars represent standard deviations. **D.** Bar charts showing the DSCAM level in each normal skin biopsy normalised by the total quantity of the normal skin biopsy protein (assayed by the Stain-Free technology) present on the membrane. The bars are coloured according to the normal skin biopsy SNP rs850678541 genotype.
(TIF)

S6 Fig Multidimensional scaling plot showing that the Guiding Eye for the Blind Dogs form a distinct cluster in Set 1. (TIF)
