# Supplementary figures and images for: A synonymous germline variant in a gene encoding a cell adhesion molecule is associated with cutaneous mast cell tumour development in Labrador and Golden Retrievers
Source: PLoS Genet. 2019 Mar 22;15(3):e1007967. doi: 10.1371/journal.pgen.1007967 (PMC6447235; doi:10.1371/journal.pgen.1007967)

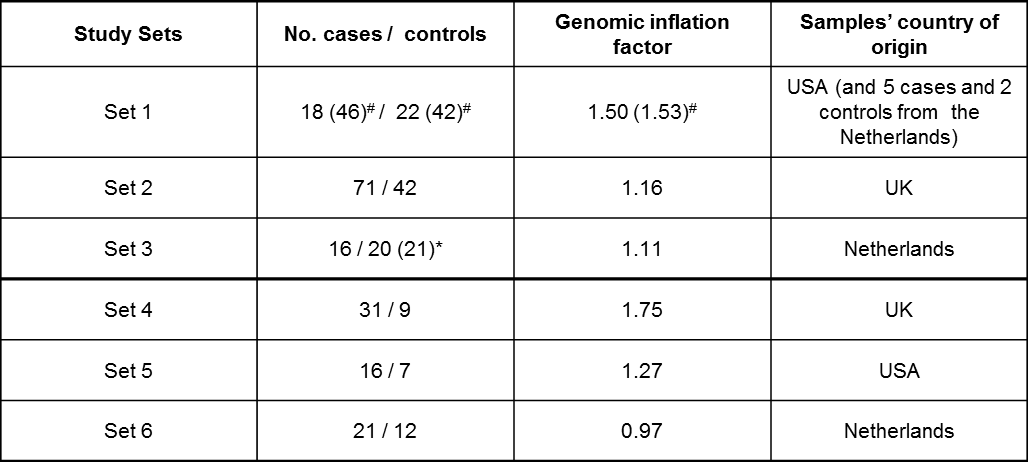

Supplement: S1 Table — #Number of cases and controls and genomic inflation factors for GWAS dataset before exclusion of Guiding Eye for the Blind Dogs. *Number of control dogs before exclusion, in the meta-analysis including Sets 1–6, of one individual that subsequent to genotyping had been reported as being affected by cancer (not MCT). (TIF) [file pgen.1007967.s001.tif]

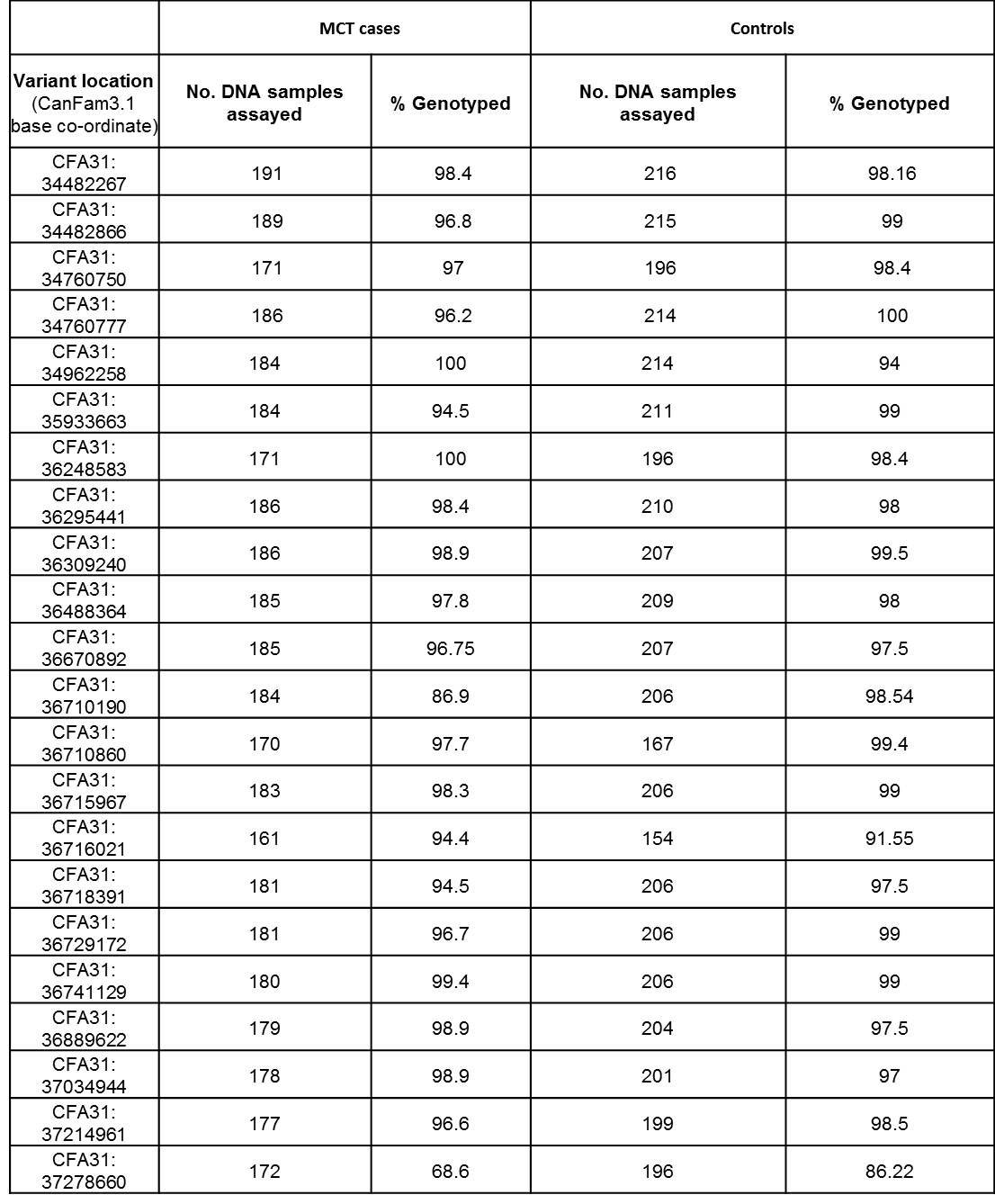

Supplement: S2 Table — The percentage of DNA samples that were successfully genotyped by each assay is indicated. (TIF) [file pgen.1007967.s002.tif]

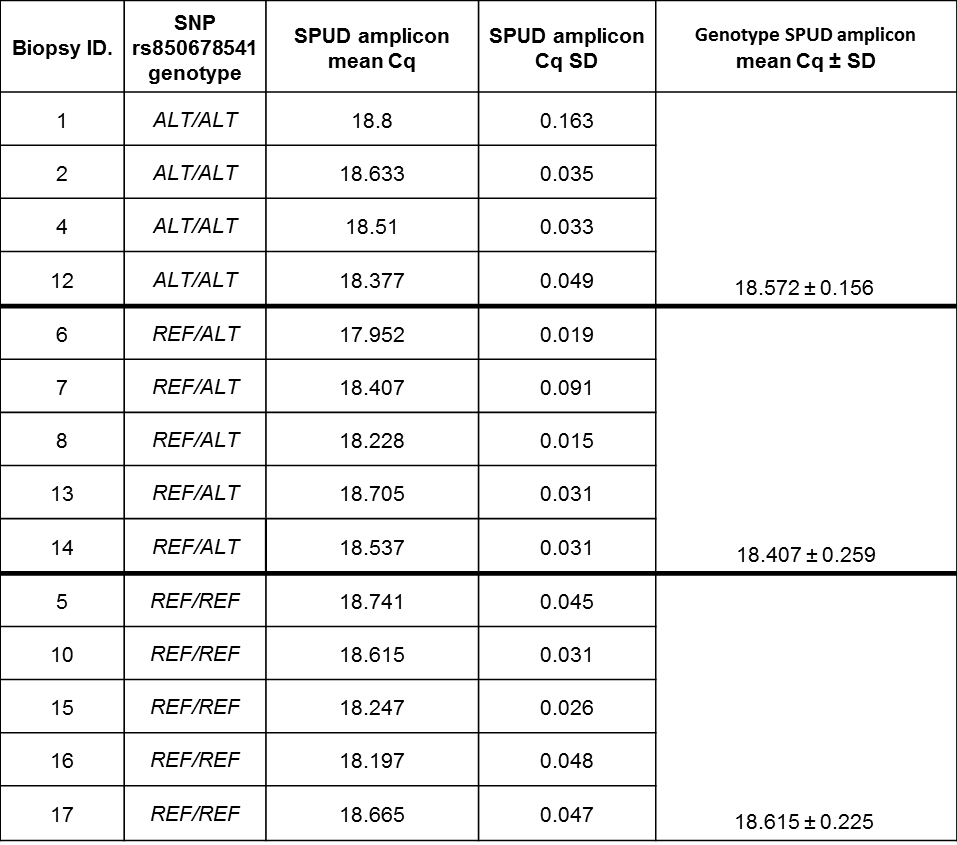

Supplement: S3 Table — SNP rs850678541 genotypes are represented by: ALT/ALT—Alternative (variant) ‘A’ allele homozygote; REF/REF—Reference ‘G’ allele homozygote; REF/ALT—GA heterozygote. (TIF) [file pgen.1007967.s003.tif]

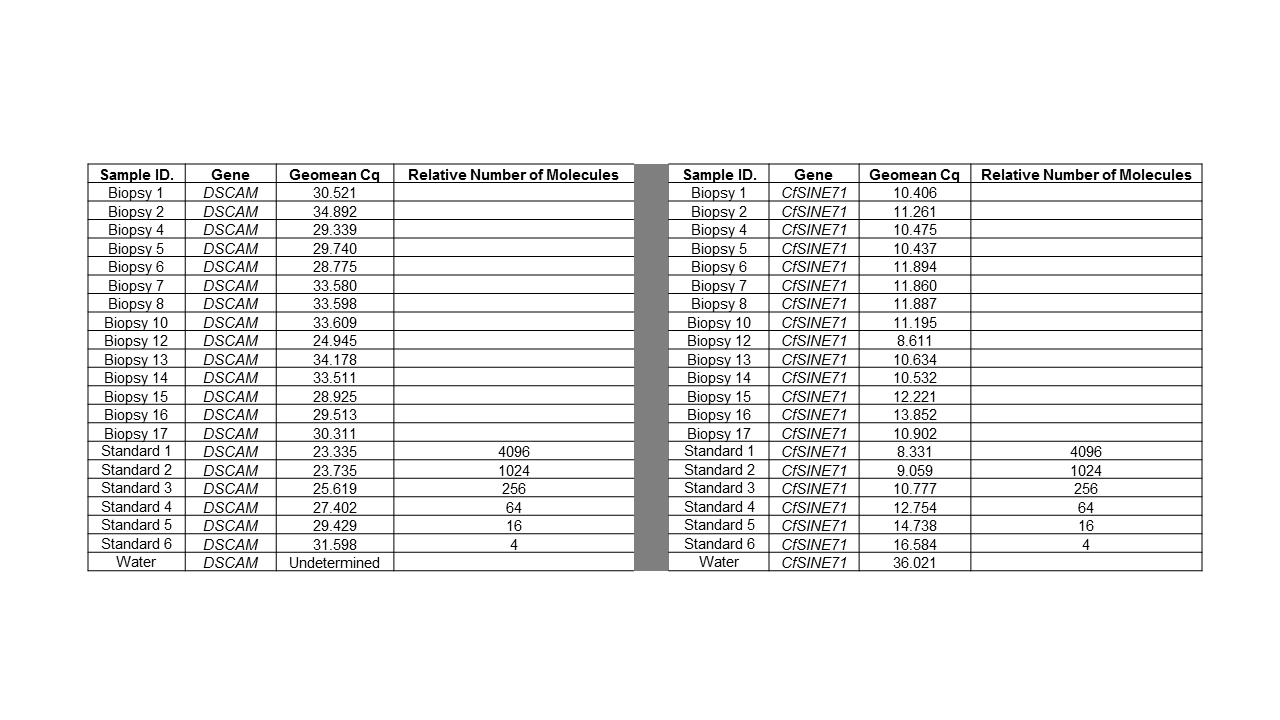

Supplement: S4 Table — (TIF) [file pgen.1007967.s004.tif]

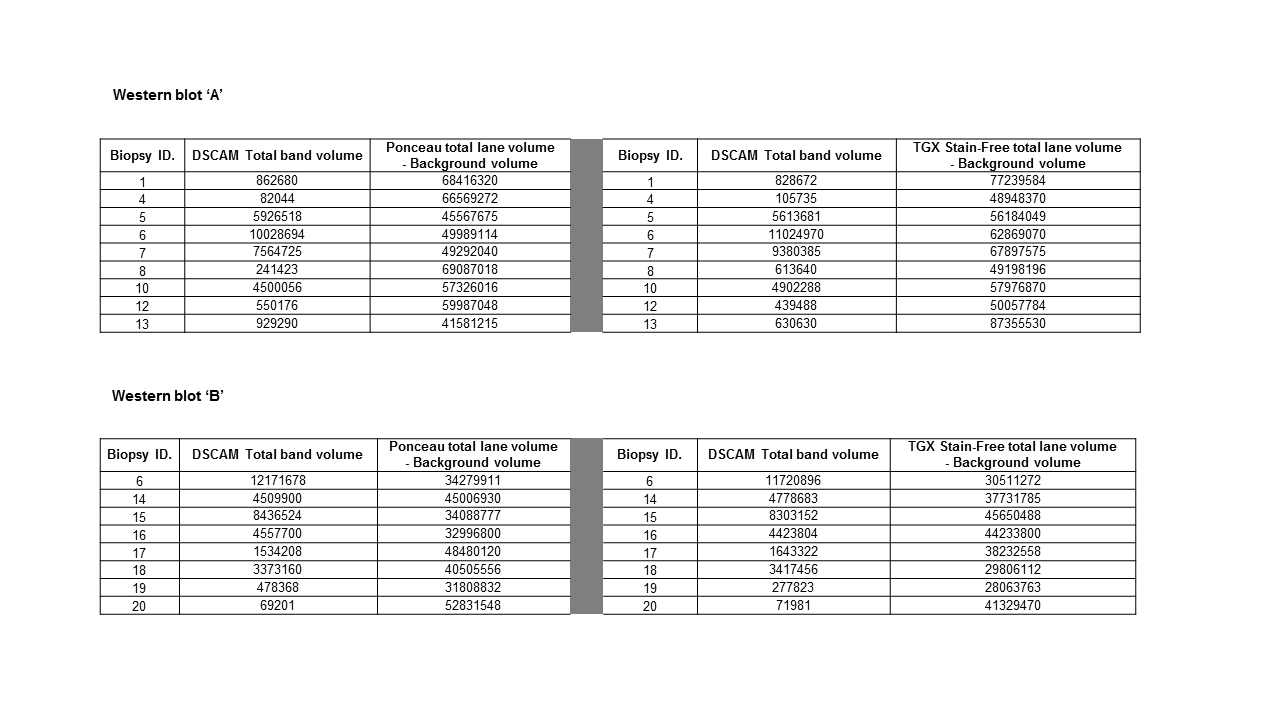

Supplement: S5 Table — (TIF) [file pgen.1007967.s005.tif]

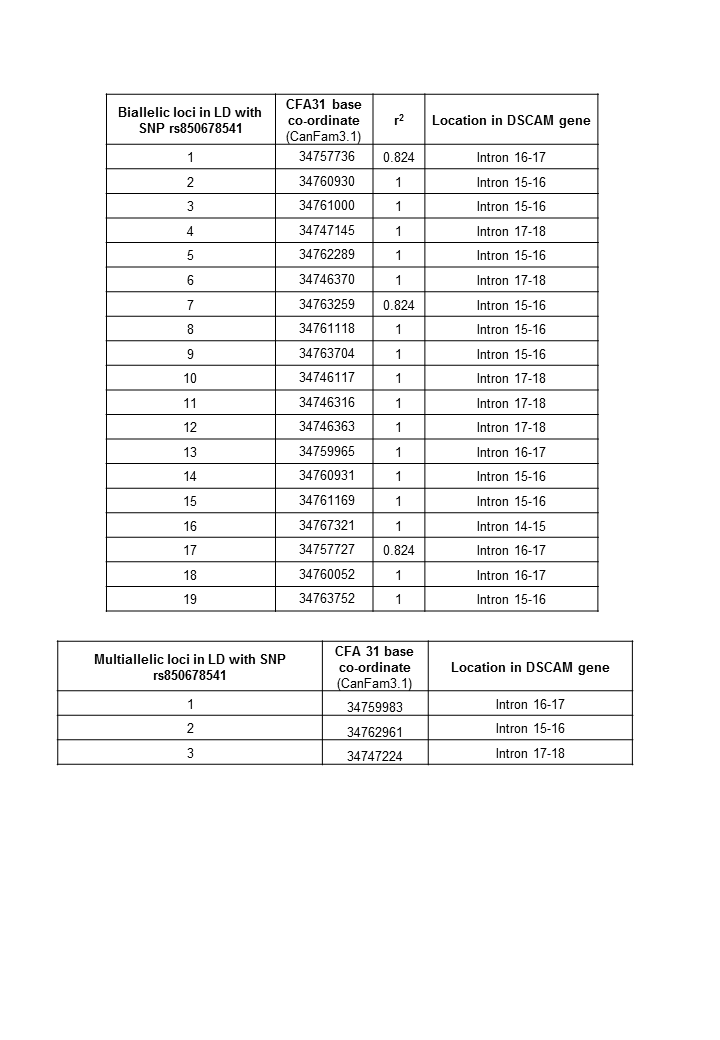

Supplement: S6 Table — The table containing the biallelic variants shows the r2 values obtained from analysis performed using the “Tagger” function of Haploview, using a r2 threshold of 0.8 and SNP rs850678541 as a tagger. The variants’ locations in the DSCAM gene are also listed. Intron 14–15—the variant is located in the intron between exons 14 and 15; Intron 15–16—the variant is located in the intron between exons 15 and 16. (TIF) [file pgen.1007967.s006.tif]

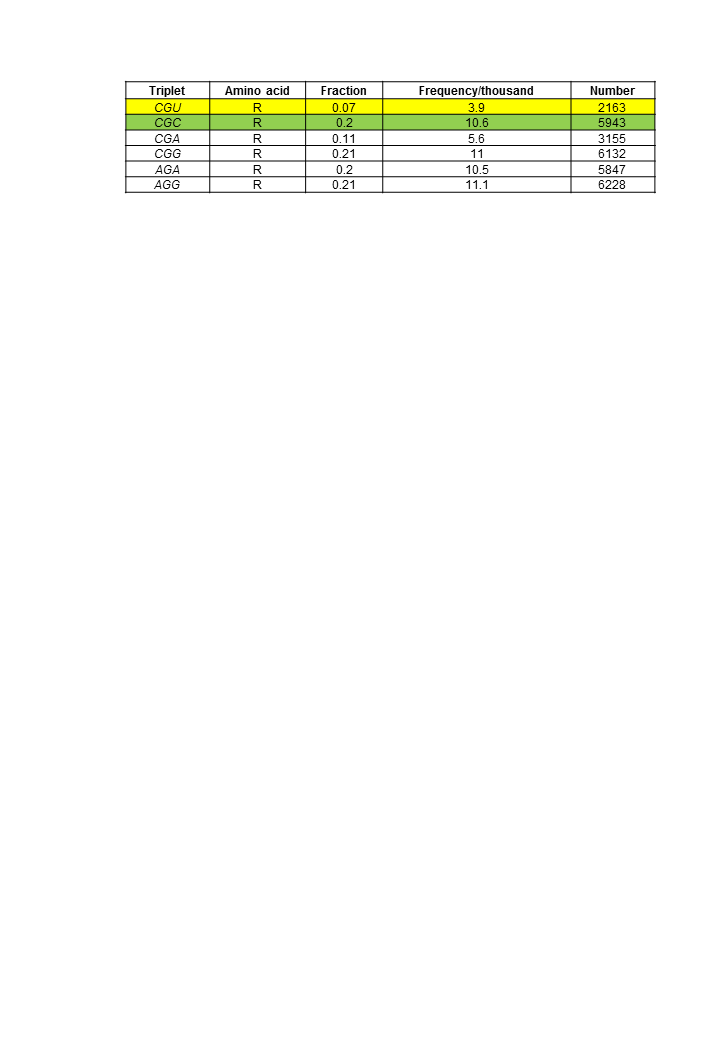

Supplement: S7 Table — Information pertaining to the reference CGC codon is highlighted in green, and to the alternative CGT codon is highlighted in yellow. (TIF) [file pgen.1007967.s007.tif]

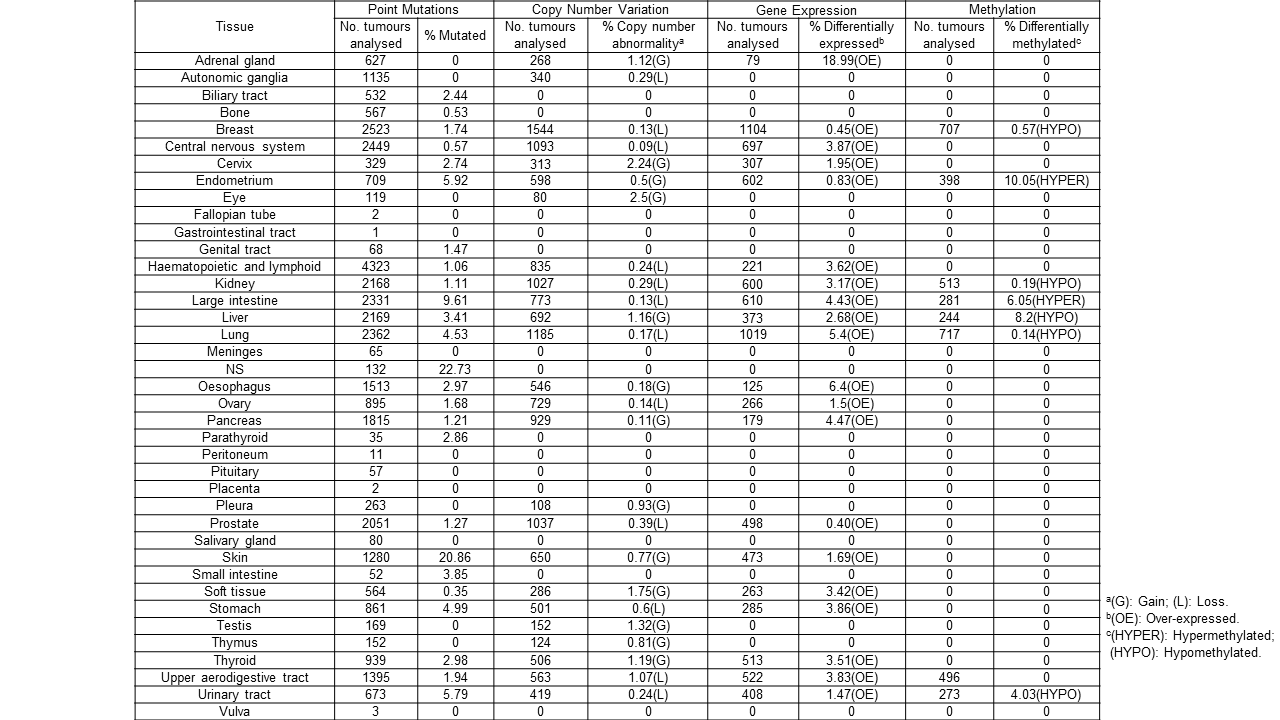

Supplement: S8 Table — Source: COSMIC database [60]. (TIF) [file pgen.1007967.s008.tif]

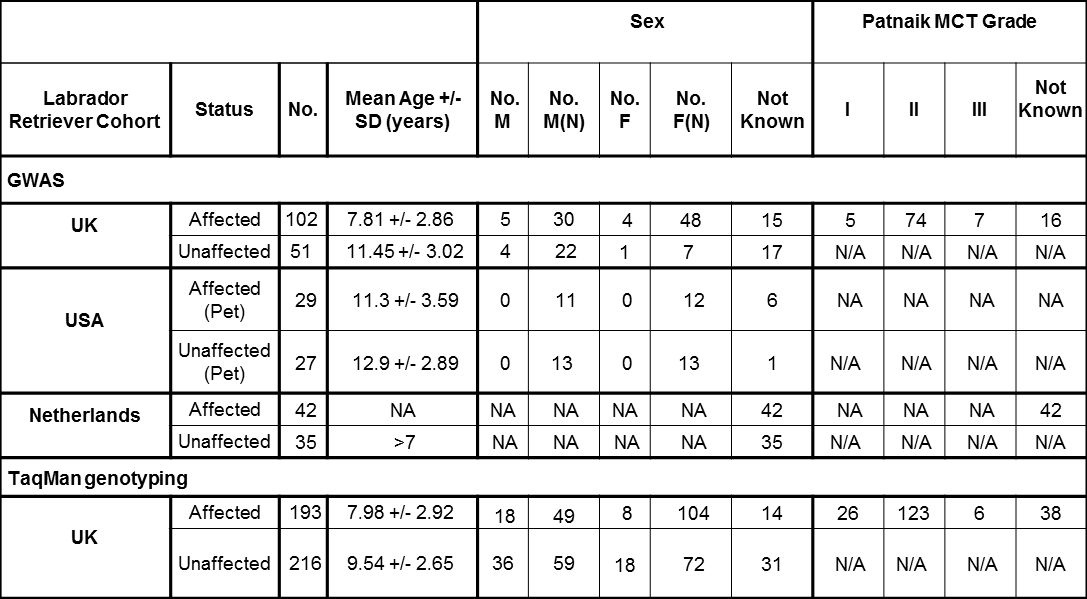

Supplement: S9 Table — M: Male; F: Female; (N): neutered; NA: Not available; N/A: Not applicable. (TIF) [file pgen.1007967.s009.tif]

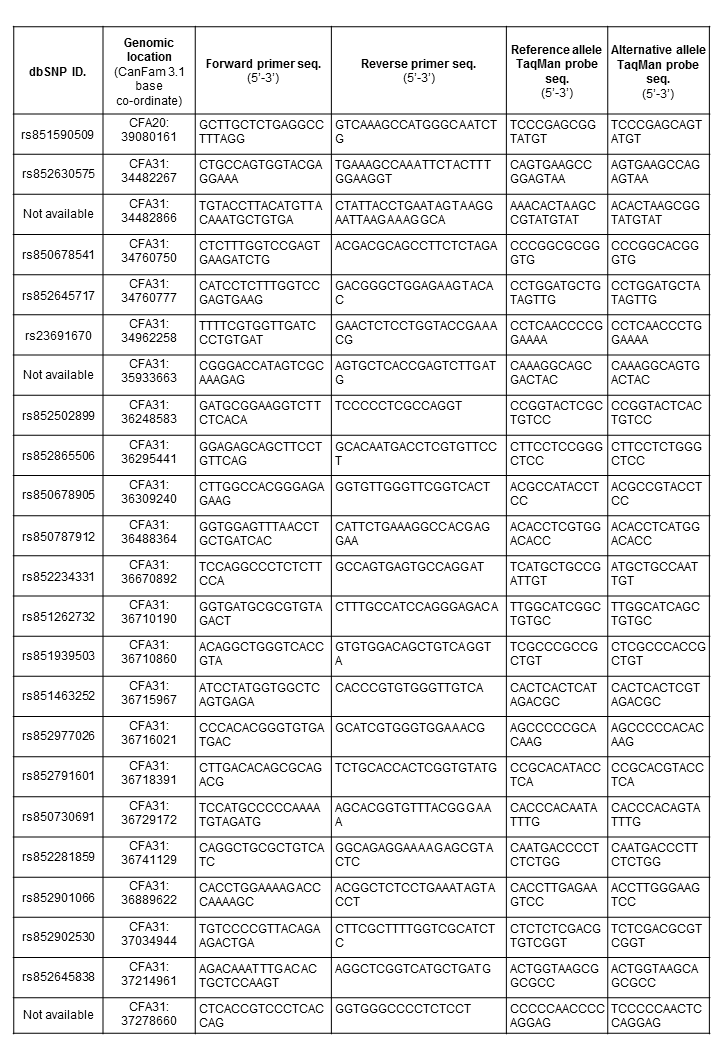

Supplement: S10 Table — (TIF) [file pgen.1007967.s010.tif]

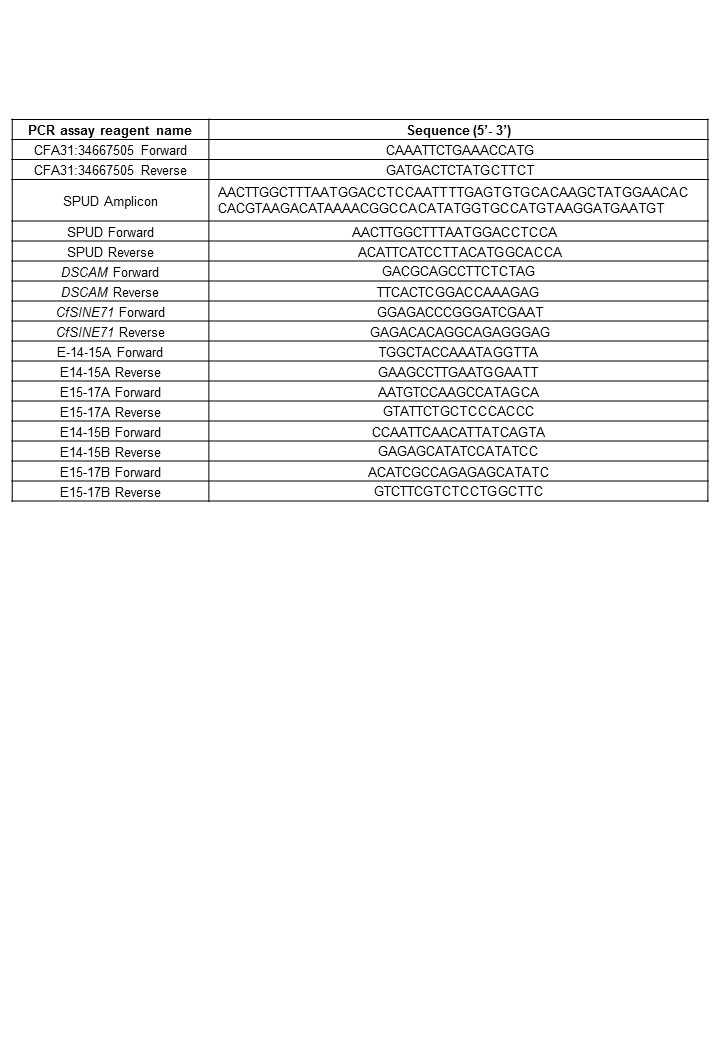

Supplement: S11 Table — (TIF) [file pgen.1007967.s011.tif]

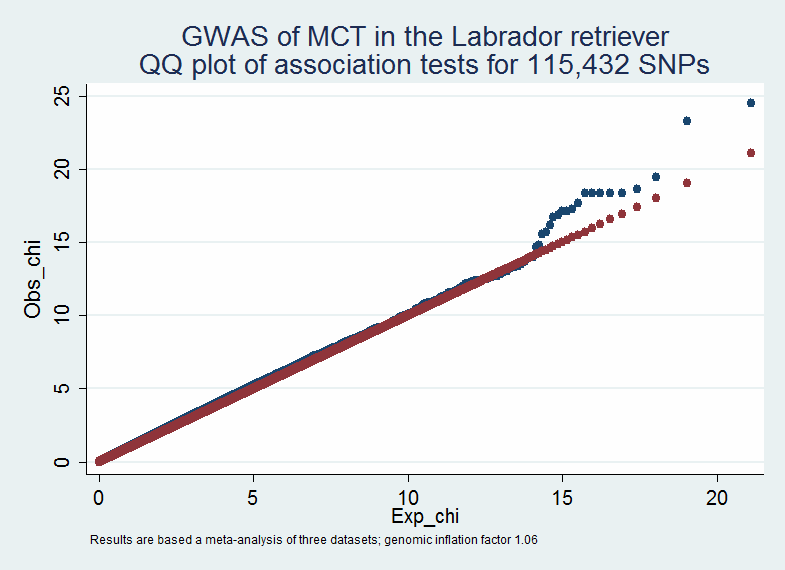

Supplement: S1 Fig — Red spots denote chi-squared values expected under the null for each of the number of SNPs tested; blue spots denote the observed chi-squared values for each SNP. (TIF) [file pgen.1007967.s012.tif]

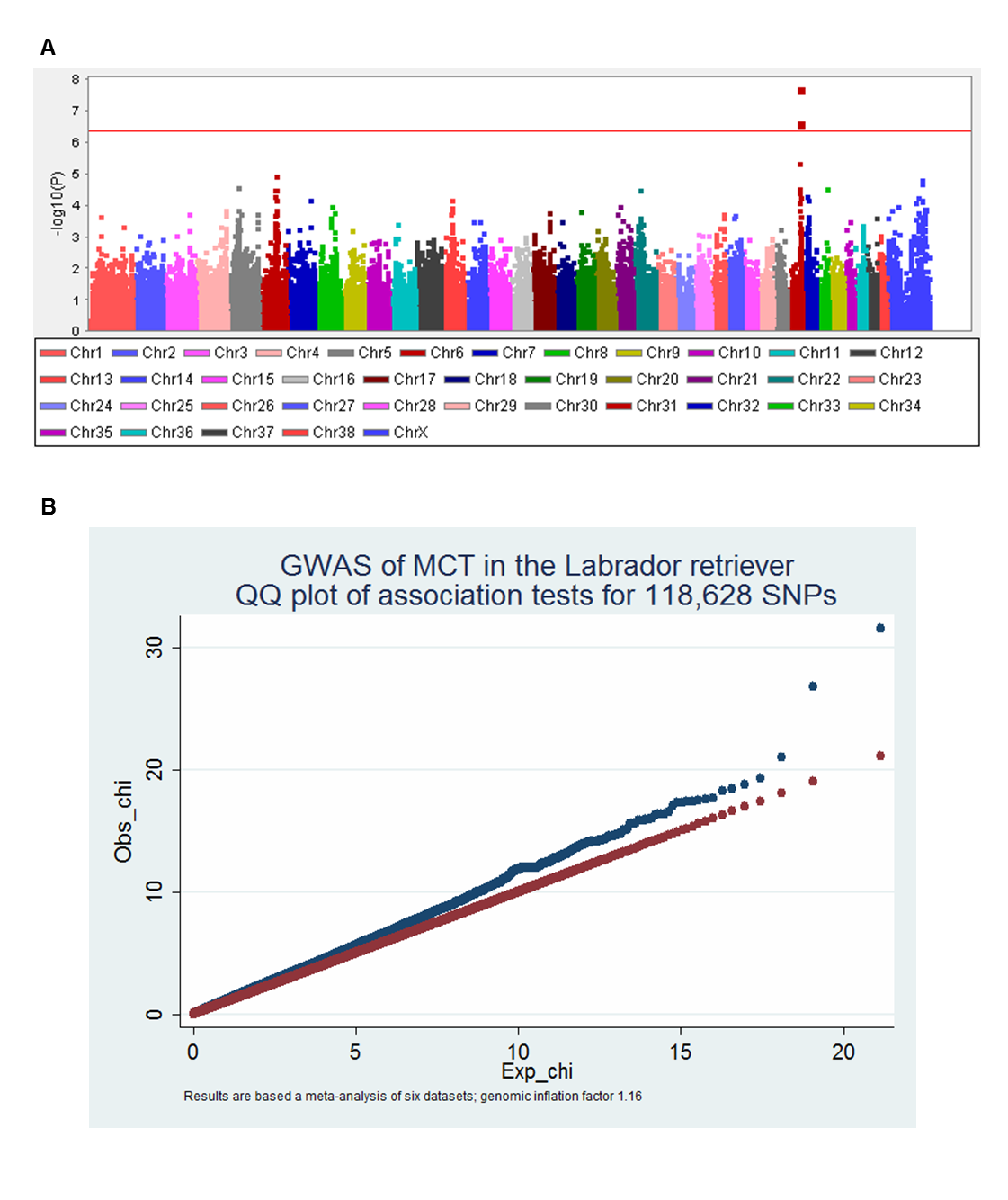

Supplement: S2 Fig — A. Manhattan plot of a combined analysis of 173 cases and 112 controls from six case-control sets. Analyses comprised 118,628 SNPs. The horizontal red line denotes the genome-wide association threshold based on Bonferroni correction for 118,628 tests (P-value = 4.2 x 10^−7^). The plot was generated using Haploview version 4.2 [74]. B. QQ plot for GWAS meta-analysis of six Labrador Retriever datasets. Red spots denote chi-squared values expected under the null for each of the number of SNPs tested; blue spots denote the observed chi-squared values for each SNP. (TIF) [file pgen.1007967.s013.tif]

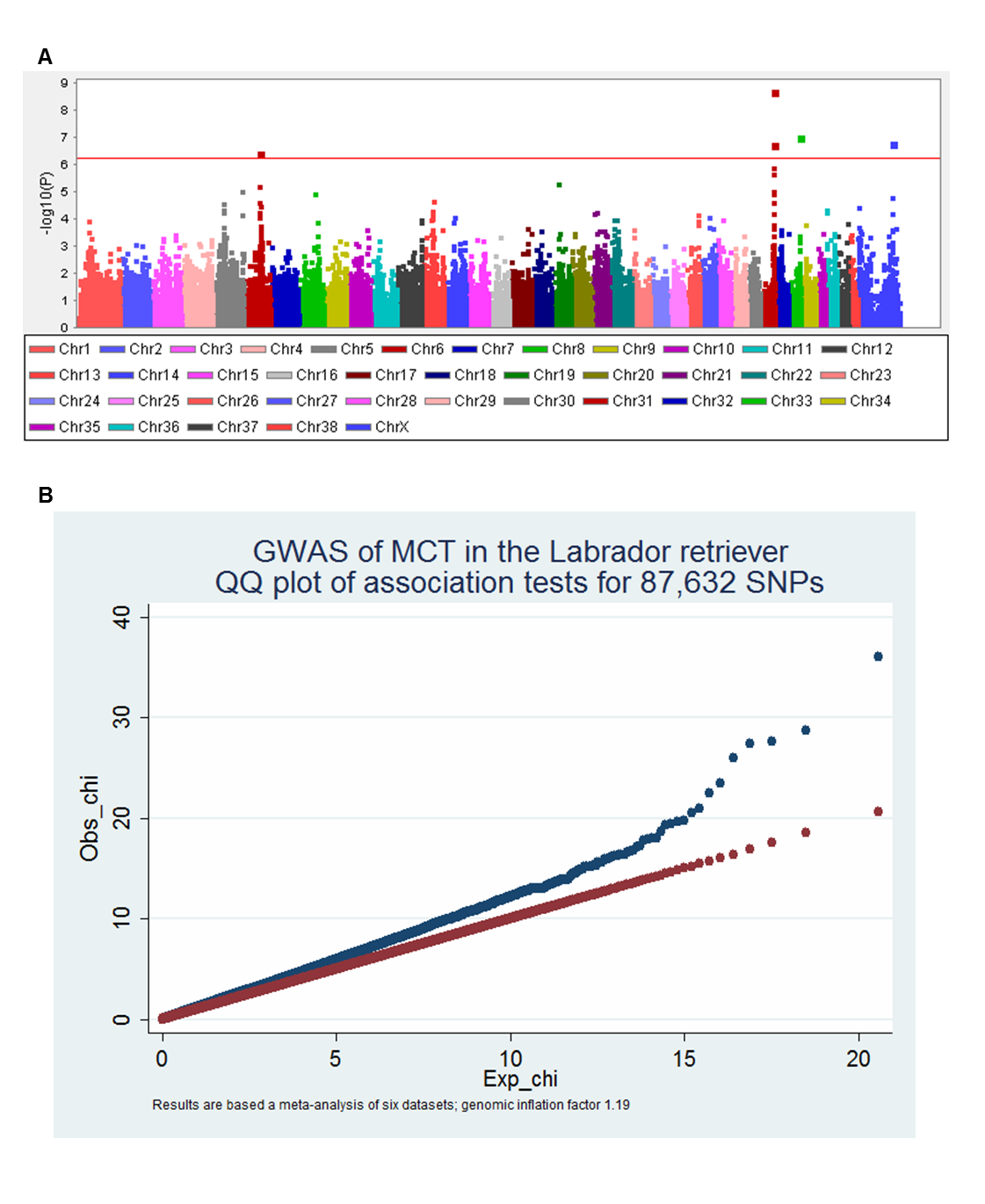

Supplement: S3 Fig — A. Manhattan plot of a combined analysis of 173 cases and 112 controls from six case-control sets. Analyses comprised 87,632 SNPs. The horizontal red line denotes the genome-wide association threshold based on Bonferroni correction for 87,632 tests (P-value = 5.7 x 10^−7^). The plot was generated using Haploview version 4.2 [74]. B. QQ plot for GWAS meta-analysis of six Labrador Retriever datasets after adjustment for population stratification. Red spots denote chi-squared values expected under the null for each of the number of SNPs tested; blue spots denote the observed chi-squared values for each SNP. (TIF) [file pgen.1007967.s014.tif]

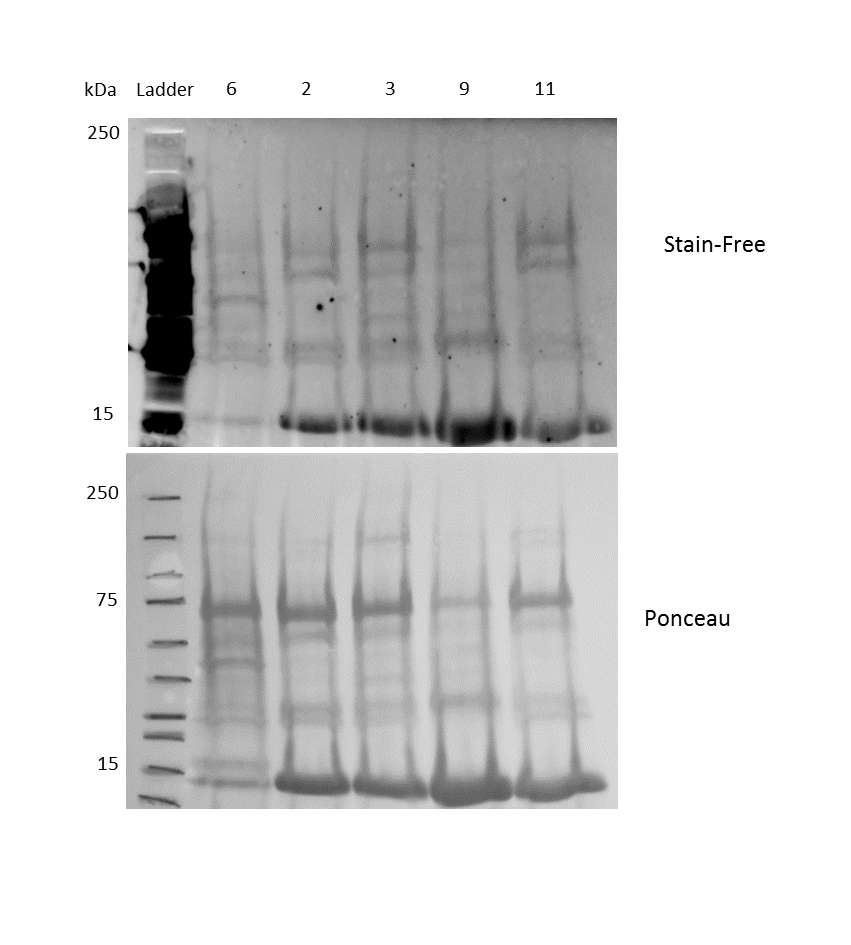

Supplement: S4 Fig — MCT biopsies #2, 3, 9 and 11 were not utilised for assay of DSCAM protein expression because the presence of an intensely staining ~15kDa band in each protein sample suggested significant protein degradation. The ~15kda band was significantly ‘weaker’ in the MCT biopsy #6 protein sample that was employed as an inter-membrane calibrator (for normalisation of DSCAM protein levels). (TIF) [file pgen.1007967.s015.tif]

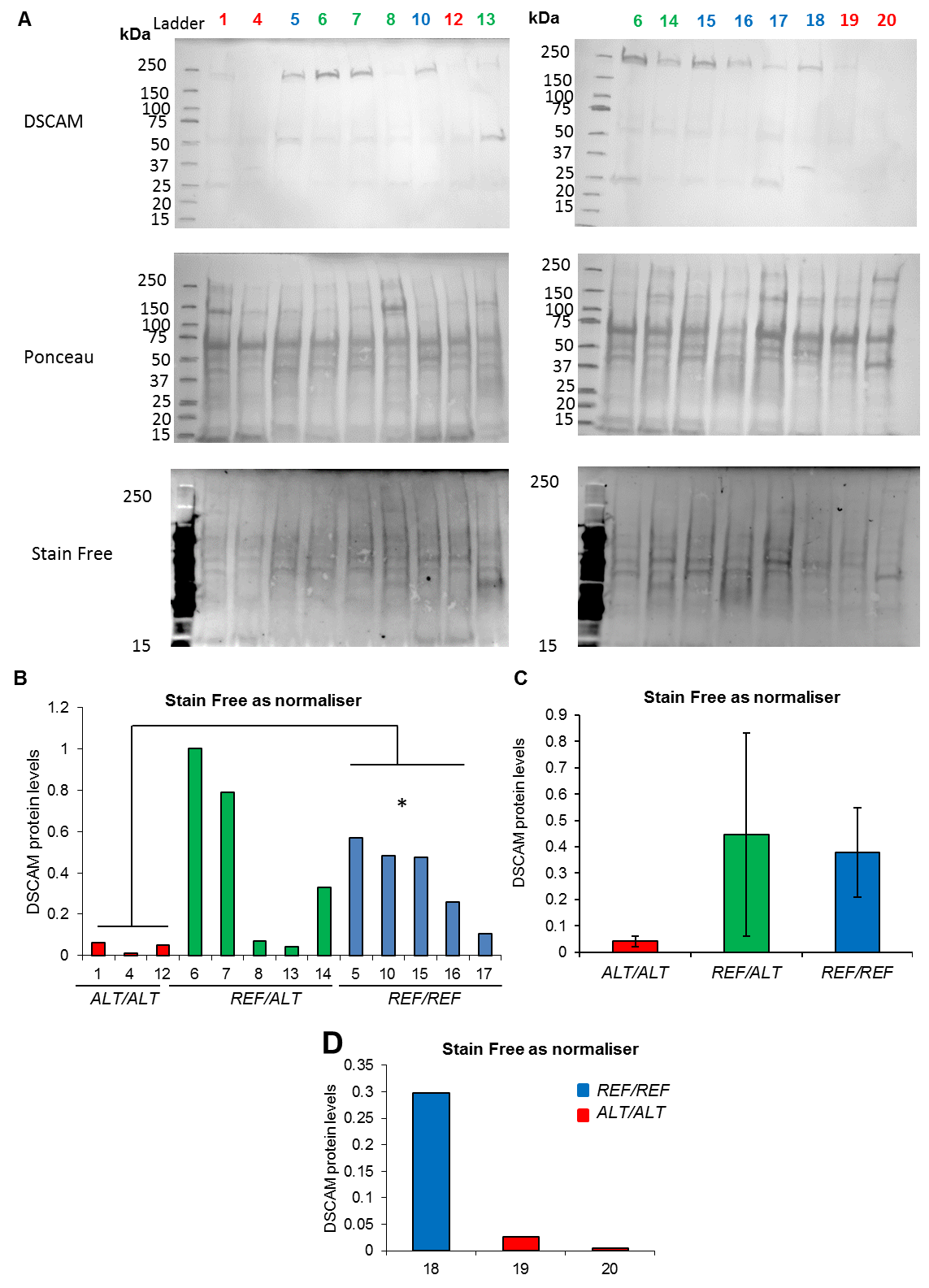

Supplement: S5 Fig — A. Whole western blot images of DSCAM antibody staining and total protein staining (through Ponceau staining and Stain-Free technology) of protein samples extracted from Labrador Retriever MCT biopsies (#1–17) and normal skin biopsies (#18–20). Sample number colours indicate SNP rs850678541 genotype: Red = Alt/Alt [Alternative (variant) ‘A’ allele homozygote]; Green = Ref/Alt [G/A heterozygote]; Blue = Ref/Ref [Reference ‘G’ allele homozygote]. B. Bar charts showing the DSCAM level in each MCT biopsy normalised by the total quantity of the MCT biopsy protein (assayed by the Stain-Free technology) present on the membrane. The biopsies are grouped according to their SNP rs850678541 genotype. *P≤0.05 (Mann-Whitney U test). C. Bar charts showing the mean DSCAM protein level +/- SD of each MCT biopsy SNP rs850678541 genotype group. Error bars represent standard deviations. D. Bar charts showing the DSCAM level in each normal skin biopsy normalised by the total quantity of the normal skin biopsy protein (assayed by the Stain-Free technology) present on the membrane. The bars are coloured according to the normal skin biopsy SNP rs850678541 genotype. (TIF) [file pgen.1007967.s016.tif]

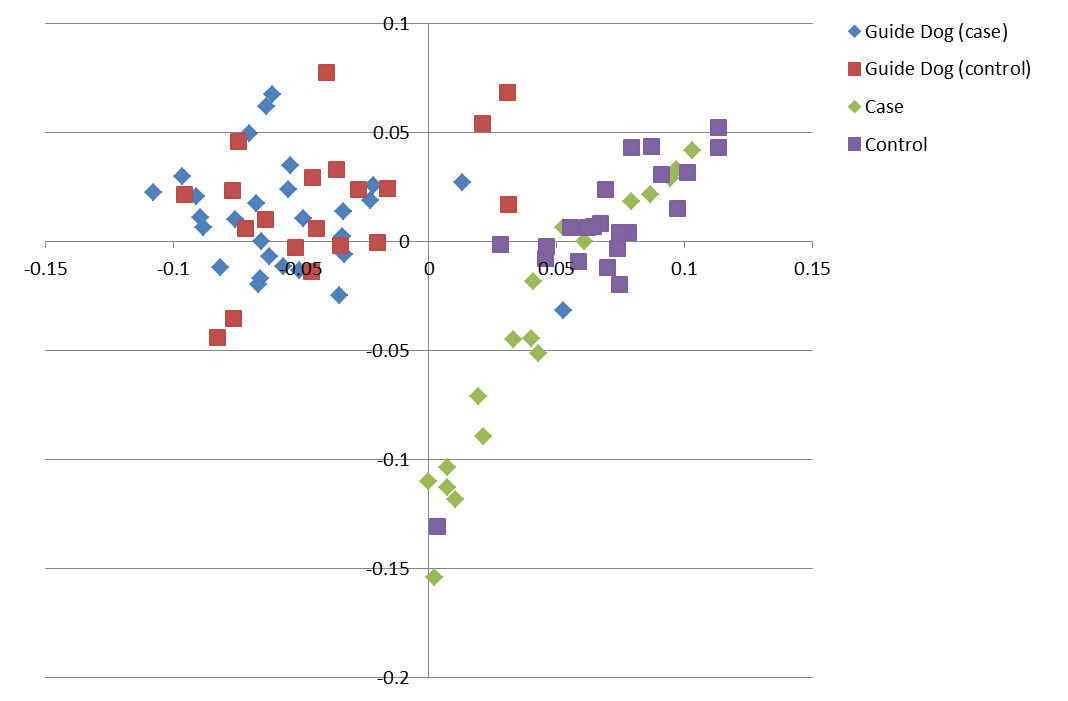

Supplement: S6 Fig — (TIF) [file pgen.1007967.s017.tif]
